# Integrative Genomic Profiling of Pediatric Solid Tumors Reveals Clinically Relevant Variants and Chromosomal Arm Aneuploidies Signatures

**DOI:** 10.1002/cam4.71666

**Published:** 2026-03-03

**Authors:** Bingxiao Yan, Jinhu Wang, Jieni Xiong, Shuangai Liu, Yinbing Tang, Ziqi He, Hujin Yan, Bize Guo, Chen Chen, Yijie Zhang, Qinfang Zhu, Jiabin Cai, Min He, Xuan Wu, Junqing Mao, Lifeng Zhang, Weizhong Gu, Zhu Zhu, Zheming Li, Rui Xiao, Qiang Shu, Gang Yu, Ting Tao

**Affiliations:** ^1^ Department of Surgical Oncology, Children's Hospital Zhejiang University School of Medicine National Clinical Research Center for Children and Adolescents' Health and Diseases Hangzhou Zhejiang China; ^2^ Biosan (Hangzhou) Clinical Laboratory Hangzhou Zhejiang China; ^3^ Child Health Innovation Research Center Binjiang Institute of Zhejiang University Hangzhou Zhejiang China; ^4^ Pediatric Cancer Research Center, National Clinical Research Center for Children and Adolescents' Health and Diseases Children's Hospital Zhejiang University School of Medicine Hangzhou Zhejiang China; ^5^ Zhejiang Key Laboratory of Neonatal Diseases Children's Hospital Zhejiang University School of Medicine Hangzhou Zhejiang China; ^6^ Cancer Center Zhejiang University Hangzhou Zhejiang China; ^7^ Department of Veterinary Medicine, College of Animal Sciences Zhejiang University Hangzhou Zhejiang China; ^8^ Department of Pathology Children's Hospital Zhejiang University School of Medicine Hangzhou Zhejiang China; ^9^ National Clinical Research Center for Children and Adolescents' Health and Diseases, National Children's Regional Medical Center Children's Hospital Zhejiang University School of Medicine Hangzhou Zhejiang China; ^10^ Sino‐Finland Joint AI Laboratory for Child Health of Zhejiang Province Hangzhou Zhejiang China

**Keywords:** chromosome arm aneuploidies, clinically relevant variants, germline predisposition variants, pediatric solid tumors

## Abstract

**Background:**

Pediatric malignancies have emerged as the leading cause of disease‐related mortality in children, exhibiting distinct etiological and molecular characteristics compared to adult cancers. Despite advances in genomic profiling, the molecular landscape of pediatric solid tumors, particularly in Chinese populations, remains undercharacterized.

**Methods:**

Through targeted next‐generation sequencing of 94 pediatric solid tumors, we systematically analyzed single nucleotide variants, short insertions/deletions, copy number variations, and chromosomal arm‐level aneuploidy, with particular emphasis on subtype‐specific genomic architectures.

**Results:**

Tumor relevant variants were identified in 74.5% of cases, comprising germline predisposition variants (17.0%) with higher prevalence in blastomas and somatic mutations (71.3%). Remarkably, 58.5% harbored therapeutic targets or guideline‐recommended biomarkers, providing molecular rationales for precision therapeutic strategies. Key findings revealed tumor‐type specific chromosomal instability patterns: sarcoma‐characteristic chromosome 8 gains, neuroblastoma‐enriched 17q amplifications, and *TP53* mutations co‐occurring with 1q gains—a profile divergent from adult malignancies.

**Conclusion:**

This study establishes the most comprehensive genomic atlas of Chinese pediatric solid tumors to date, delineating subtype‐specific oncogenic variants and chromosomal instability signatures. Our findings advance the understanding of childhood cancer pathogenesis and provide a framework for molecularly guided clinical decision‐making.

## 1 Introduction

Pediatric malignancies have emerged as the leading cause of disease‐related mortality in children [1]. According to data reported by the World Health Organization (WHO), approximately 400,000 children and adolescents aged 0–19 years worldwide are diagnosed with cancer annually [1]. Recent epidemiological data from China revealed that a total of 121,145 pediatric and adolescent cancer cases were documented between 2018 and 2020, with an average annual incidence of 40,381 newly diagnosed cases [2]. Regarding tumor type distribution, leukemias accounted for the largest proportion (approximately 32%), followed by lymphomas (9.7%), while solid tumors collectively represented the majority of cases, constituting nearly 60% of the total—a prevalence exceeding the previously estimated proportion of less than half and highlighting the need for further investigation [1, 2].

Pediatric tumors exhibit fundamental biological and clinical distinctions from adult malignancies, with profound implications for tumorigenesis mechanisms and therapeutic decision‐making. First, the tumor spectra demonstrate marked divergence: childhood cancers are predominantly characterized by hematopoietic malignancies (e.g., leukemias), central nervous system tumors, lymphomas, neuroblastomas, and osteosarcomas, whereas adult malignancies primarily comprise epithelial‐derived carcinomas such as gastrointestinal cancers, lung cancer, and breast cancer [3]. Second, genomic mutational burden displays significant age‐related disparities. Although mutation frequencies in pediatric tumors vary across histological subtypes (0.02–0.49 mutations/Mb), their overall mutational load is merely 1/14th that of adult cancers (0.13 vs. 1.8 mutations/Mb) [4]. Third, molecular pathology reveals intrinsic differences: a genomic analysis of 961 tumors identified only 30% overlap in significantly mutated genes between pediatric and adult cohorts, while another study of 1699 tumors further demonstrated merely 45% concordance in driver genes [4, 5]. Notably, the overall 5‐year survival rate for pediatric malignancies exceeds 80%, reflecting substantially superior clinical outcomes compared to adult counterparts [6].

The rapid advancement and widespread adoption of next‐generation sequencing (NGS) technologies have enabled high‐throughput genomic profiling of tumor specimens [3, 4, 5, 7]. Characterized by comprehensive variant detection capacity and single‐nucleotide resolution, NGS has evolved into a cornerstone of precision oncology in pediatric malignancies, driven by continuous technological refinements and bioinformatics algorithm optimization [8, 9, 10, 11]. Previous studies have documented NGS‐based genomic landscapes in multiple pediatric cancer cohorts, including 961 tumors (Nature, 2018), 1699 tumors (Nature, 2018), 309 patients (Cancer Discovery, 2021), and 282 tumors (Nature Medicine, 2024) [4, 5, 12, 13].

Although existing pediatric cancer cohort studies encompass diverse tumor types, there persists a notable research bias with hematologic malignancies predominating in published datasets [4, 5, 12, 13]. Molecular characterization of solid tumors remains insufficiently explored, particularly regarding the genomic landscape of pediatric solid tumors in Chinese populations, which remains systematically underexplored. To address this, we prospectively collected 94 pediatric solid tumor samples with matched peripheral blood controls from Children's Hospital Zhejiang University School of Medicine. Utilizing targeted capture‐based NGS and an established bioinformatics pipeline, we systematically identified single nucleotide variants (SNVs), short insertions/deletions (InDels), copy number alterations (CNAs), and structural variations, followed by integrative analysis of germline and somatic mutation patterns across distinct histopathological subtypes.

## 2 Materials and Methods

### 2.1 Study Design and Patients

In this study, we prospectively enrolled 94 patients with pediatric solid tumors from Children's Hospital Zhejiang University School of Medicine. In this study, the types of pediatric tumors involved are primarily focused on sarcomas and neuroblastomas, due to the limitation of sample sources to the admission conditions of specific hospitals. In addition, a small number of other types, such as liver and kidney tumors, are also included. The cohort represents a consecutive series of cases for which paired tumor and blood samples were available, aiming to reflect the real‐world spectrum of disease severity and stages encountered at our tertiary care center. It should be noted that while the cohort includes patients across various disease stages, a detailed analysis of the correlation between tumor stage and germline variant prevalence was beyond the primary scope of this foundational genomic profiling study. These samples were subsequently utilized for the extraction of genomic DNA and high‐throughput sequencing analysis. This study was approved by the Ethics Committee of Children's Hospital, Zhejiang University School of Medicine (2020‐IRB‐049 and 2024‐IRB‐0191‐P‐01).

### 2.2 Library Construction and Sequencing

Genomic DNA extraction from peripheral blood and fresh tissue was performed using the MagaBio Plus Universal Genomic DNA Purification Kit (Bioer, Hangzhou, Zhejiang, China), while genomic DNA extraction from formalin‐fixed paraffin‐embedded (FFPE) samples was carried out using the FastPure FFPE DNA Isolation Kit (Vazyme, Nanjing, Jiangsu, China). The genomic DNA was sheared using the Covaris LE200 instrument (Covaris, Woburn, MA, USA). Sequencing libraries were prepared using the VAHTS Universal DNA Library Prep Kit for Illumina V3 (Vazyme, Nanjing, Jiangsu, China) through a series of steps, including end repair, A‐base addition, ligation of Illumina sequencing adaptors, followed by PCR amplification, and clean‐up. The synthesis of panel probes was completed by iGeneTech Biotechnology Co. Ltd. (Jiaxing, Zhejiang, China). The panel was designed to target coding regions or mutational hotspots of key genes, as well as recurrent non‐coding regions associated with gene fusions, collectively covering 1.39 Mb of sequence across 481 genes. The list of panel genes with annotated covered regions is presented in Table S2. Targeted region capture was performed using the TargetSeq One Hyb & Wash Kit v2.0 (iGeneTech, Jiaxing, Zhejiang, China), through several steps, including probe hybridization, capture and washing with streptavidin‐conjugated beads, followed by PCR amplification, and clean‐up. The pooled libraries containing the captured DNA fragments were subsequently sequenced on the MGI DNBSEQ‐T7 platform (MGI, Shenzhen, Guangzhou, China), generating 2 × 150‐bp paired‐end reads with a minimum data output of 5 Gb to ensure a mean coverage depth of no < 1500× across the targeted regions.

### 2.3 Sequence Data Alignment and Variant Detection

In this study, we employed fastp (version 0.23.4) for quality control of the raw sequencing data, which included assessing data quality, removing low‐quality sequences, and eliminating adapter sequences. Subsequently, minimap2 (version 2.26) was utilized to align the high‐quality, filtered data to the human genome (hg19), generating BAM format files. Thereafter, GATK (version 4.4.0.0) and vardict (version 1.8.2) were jointly applied for the detection and analysis of SNVs and InDels. Somatic variants were reported only if they reached a variant allele frequency (VAF) threshold of ≥ 5%. Fusion gene detection was performed using Lumpy (version 0.2.13) and GeneFuse (version 0.8.0). The detection results were manually verified using the Integrative Genomics Viewer (IGV).

In this study, the detection of gene copy numbers referenced the method reported by Donovan T. Cheng [14]. Copy number aberrations were identified by comparing the sequence coverage of targeted regions in tumor samples with that of standard diploid normal samples. Specifically, the GATK DepthOfCoverage tool was used to calculate the coverage of targeted regions. The average sequencing depth of the target regions in the sample was divided by the overall average sequencing depth of the sample, and the sequencing depth of the target regions in all tested samples was normalized to eliminate the impact of varying sequencing data volumes on the analysis. The ratio of the normalized sequencing depth of the target regions in the sample to be tested to that in the control sample was calculated. For peripheral blood samples, a reference set composed of multiple normal peripheral blood samples was used as the control, while for tumor samples, the paired peripheral blood sample data was used as the control. A copy number below 1.3 in the sample was considered indicative of a copy number deletion, and below 0.5 was considered indicative of a homozygous deletion; a copy number above 3.8 was considered indicative of a copy number duplication, and genes with more than 8 copies were considered to have undergone gene amplification.

The majority of regions on the chromosomes are distributed with target detection areas; hence, we detect large‐scale copy number variations (CNVs) on chromosomes by analyzing the copy number changes of multiple genes within chromosomal arm segments. CNVs are identified by mapping the copy numbers of each region to the chromosomal band positions to recognize chromosomal aneuploidy. A chromosomal arm is classified as having aneuploidy if there are continuous regions on the arm showing deletions or duplications.

### 2.4 Variant Annotation and Interpretation

Multiple authoritative databases were utilized to annotate and interpret the variant results. The databases included in this analysis are COSMIC (http://cancer.sanger.ac.uk/cosmic), ClinVar (http://www.ncbi.nlm.nih.gov/clinvar/), OncoKB (https://www.oncokb.org/), gnomAD (https://gnomad.broadinstitute.org/), and ClinGen (https://www.clinicalgenome.org/). The pathogenicity of variants was assessed according to the standards for the classification of pathogenicity of somatic variants in cancer (oncogenicity), as well as established pathogenic results from ClinVar, OncoKB, ClinGen, and large cohort studies. To ensure the accuracy of the classification of P/LP (likely pathogenic/pathogenic) variants, this study included only the following two categories of variants as P/LP: (1) variants explicitly classified as P/LP in ClinVar, OncoKB, and relevant literature; (2) novel variants in tumor suppressor genes leading to splicing variants, premature termination, or other protein function loss. Regarding CNVs, this study focused solely on copy number deletions in tumor suppressor genes and copy number duplications in oncogenes, with the requirement that the duplicated oncogenes include the complete coding sequence (CDS) region.

To determine whether the identified variants in our cohort represent known mutational hotspots, we quantified the recurrence of variants with identical amino acid changes using two authoritative resources: the COSMIC Cancer Mutation Census (https://cancer.sanger.ac.uk/cmc/home, v103) and the Cancer Hotspots database (https://www.cancerhotspots.org).

To evaluate the clinical applicability of sequencing findings, we systematically categorized clinically actionable variants into two distinct classes: (1) Guideline‐recommended variants: Alterations in genes explicitly mandated for testing by NCCN guidelines specific to corresponding tumor types; (2) Therapeutically targetable variants: Variants annotated in OncoKB with FDA‐approved or investigational targeted therapies, supported by evidence‐based clinical actionability.

### 2.5 Data Analysis

Probabilistic modeling of pairs of Chromosomal Arm‐level Aneuploidy co‐occurring in the same tumor sample was based on a previously described model [15, 16, 17]. This model is summarized in Equation (1).

(1) $$p=\frac{\left(\begin{array}{c} T\\ k \end{array}\right)\times \left(\begin{array}{c} T-k\\ N2-k \end{array}\right)\times \left(\begin{array}{c} T-N2\\ N1-K \end{array}\right)}{\left(\begin{array}{c} T\\ N2 \end{array}\right)\times \left(\begin{array}{c} T\\ N1 \end{array}\right)}$$

Herein, *p* is the probability that Chromosomal Arm‐level Aneuploidy co‐occur in *k* samples out of a total number of *T* samples, given that Chromosomal Arm‐1 occurs in *N*1 samples and Chromosomal Arm‐2 occurs in *N*2 samples. Thus, the model accounts for the frequencies of each of the two individual Chromosomal Arm‐level Aneuploidy, as well as the total cohort sample size. The results were considered statistically significant when *p* ≤ 0.05.

## 3 Results

### 3.1 Characteristics of the Patients

**FIGURE 1:**
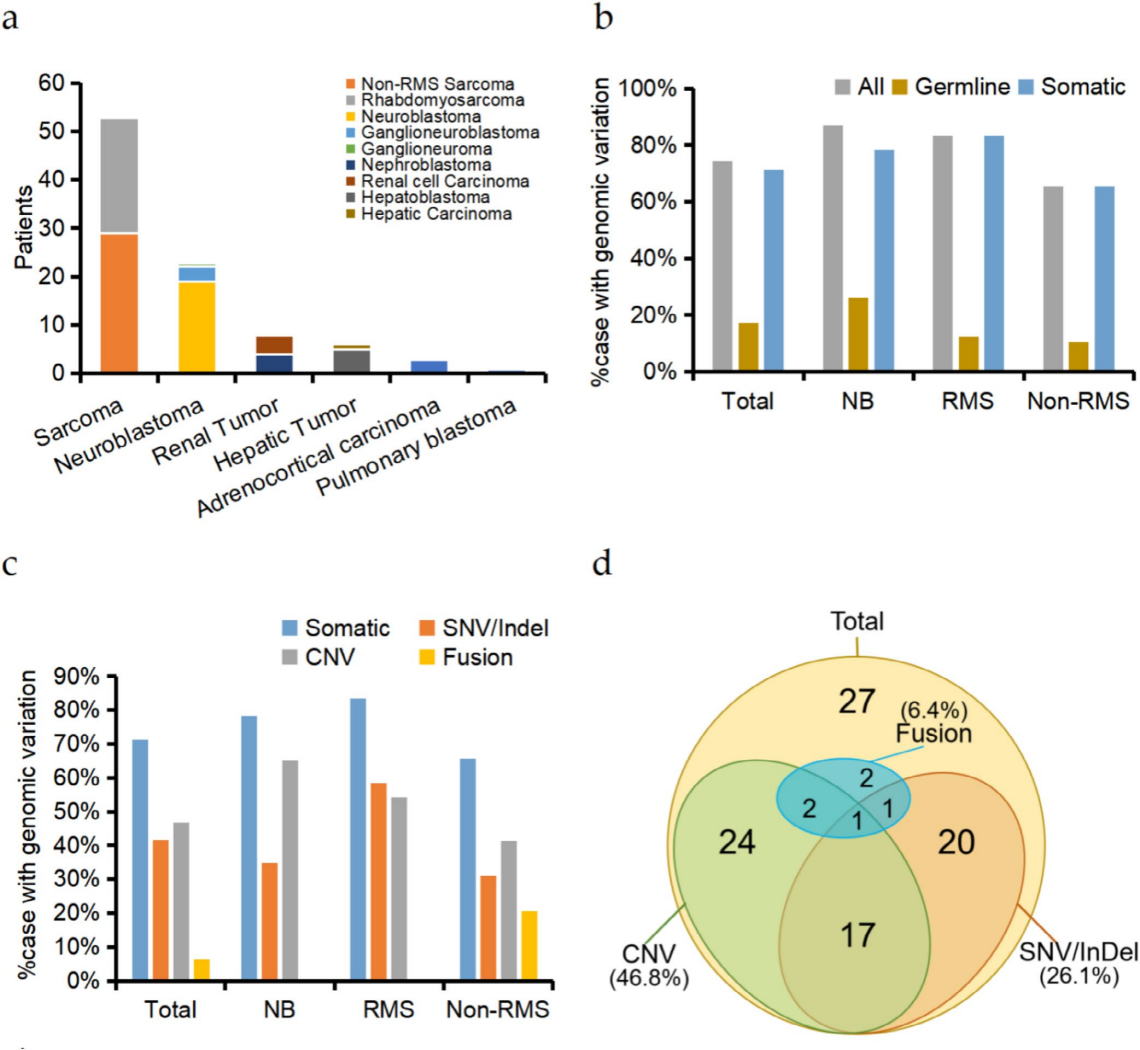
Characteristics of pediatric solid tumors and variants. (a) Diagnosis types of pediatric solid tumors, with more detailed classifications for sarcomas, neuroblastomas, renal tumors, and hepatic tumors. (b) Frequencies of variant detection in solid tumors. “All” represents all patients with detected germline or somatic variants. (c) Frequencies of different variant types detectFed in tumors. (d) Number of cases with different somatic variant types detected in tumors. NB, neuroblastoma; non‐RMS, non‐rhabdomyosarcoma; RMS, rhabdomyosarcoma.

This study enrolled 94 pediatric tumor samples for comprehensive genomic profiling (Figure 1a, Table S1). The histopathological distribution comprised: sarcomas (*n* = 53, 56.4%), including 24 rhabdomyosarcomas (RMS) and 29 non‐rhabdomyosarcomas (Non‐RMS) encompassing 3 fibrosarcomas, 2 Ewing sarcomas, 1 case each of lymphangioma, neurofibroma, and malignant peripheral nerve sheath tumor, with 21 other soft tissue sarcomas; neuroblastomas (NB) (*n* = 23, 24.5%) consisting of 19 typical neuroblastomas, 3 ganglioneuroblastomas, and 1 ganglioneuroma. Additional cases included 8 renal tumors (4 nephroblastomas and 4 renal cell carcinomas), 6 hepatic tumors (5 hepatoblastomas and 1 hepatocellular carcinoma), 3 adrenocortical carcinomas, and 1 pulmonary blastoma.

### 3.2 Mutation Frequencies Across Tumor Types

**FIGURE 2:**
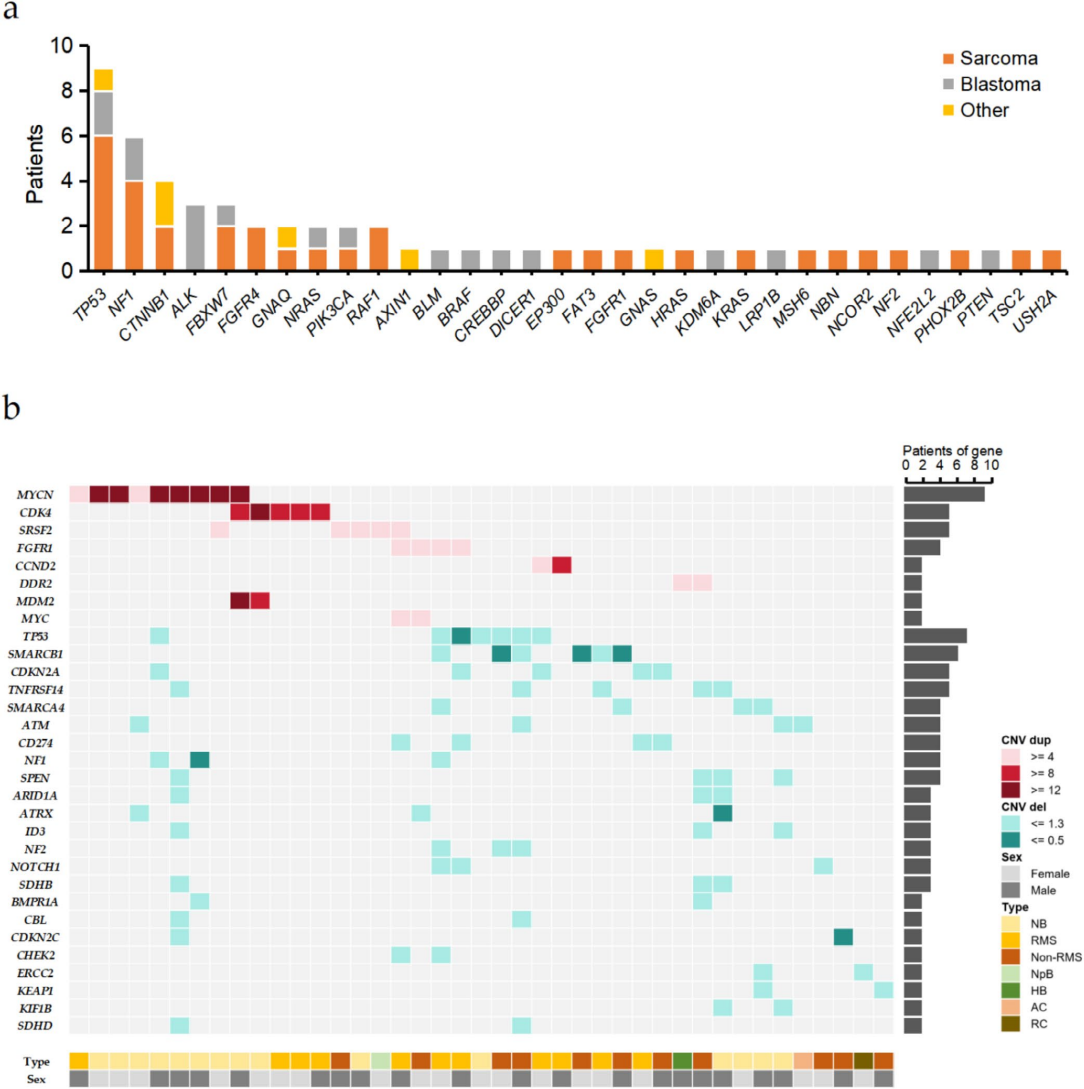
Somatic mutations in the cohort. (a) Genes containing pathogenic or likely pathogenic SNV/InDel sites. Case count for each category is shown in the same color as the legend. (b) Recurrently mutated genes containing CNV variants. Red represents gene copy number duplication (dup), while green indicates gene copy number deletions (del). The classification of gene copy number is detailed in the legend. The number of variants identified per gene is represented to the right. Tumor type and sex are represented across the bottom. AC, adrenocortical carcinoma; HB, hepatoblastoma; NB, neuroblastoma; non‐RMS, non‐rhabdomyosarcoma; NpB, nephroblastoma; RC, renal cell carcinoma; RMS, rhabdomyosarcoma.

Targeted gene panel sequencing of tumor tissues and matched peripheral blood samples revealed tumor‐related variations in 74.5% (70/94) of cases, with germline variants detected in 17.0% (16/94) and somatic alterations identified in 71.3% (67/94) (Figure 1b). The somatic mutation spectrum demonstrated: SNVs/InDels in 41.5% (39/94), CNVs in 46.8% (44/94), and gene fusions in 6.4% (6/94) of patients (Figure 1c,d). Strikingly, neuroblastomas exhibited significantly higher CNVs detection rates compared to SNVs/InDels (65.2% vs. 34.8%, *p* = 0.02 by Fisher's exact test), predominantly driven by *MYCN* amplifications (*n* = 8) (Figures 1c and 2b, Figure S1). All fusion events were exclusively observed in the Non‐RMS sarcoma subgroup.

### 3.3 Germline Variants in Cancer Predisposition Genes

We performed targeted sequencing on tumor tissues and matched peripheral blood samples to analyze germline variants. To address the heterogeneity in gene panels across existing studies, we integrated three authoritative resources: the COSMIC Cancer Gene Census (CGC) database, the MSK‐IMPACT clinical panel, and genes reported in the G4K study, resulting in a 164‐gene panel (Figure S3, Table S2) [13, 18, 19]. Germline variants were classified as P/LP based on: (1) ClinVar annotations; (2) “Oncogenic” or “Likely Oncogenic” designations in OncoKB; (3) somatic oncogenicity criteria established by Horak et al. (2022) [19, 20].

Sixteen patients (17.0%) harbored P/LP germline variants, with recurrent mutations in *NF1* (*n* = 3), *TP53* (*n* = 2), and *VHL* (*n* = 2) (Figure 1b, Table 1). These variants showed significant enrichment in tumor suppressor genes (TSG) (15 vs. 1 oncogene, *p* < 0.001 by Fisher's exact test), with *AR* being the proto‐oncogene affected. Except for a *FANCA* exon loss, all variants were SNVs, including 8 (50%) truncating alterations caused by frameshift, stop‐gain, or splice‐site mutations. Notably, 7/8 (87.5%) SNVs exhibited C>T (G>A) transitions, aligning with the characteristic signature of mutations [4].

**TABLE 1:** Detailed information on germline mutations detected in the cohort.

Sample ID	Sex	Cancer type	Gene	Gene role	HGVS nomenclature	Protein change	Variant types	Cancer Hotspots/COSMIC_Recurrence
ZJUCH_08	Male	Rhabdomyosarcoma	*DICER1*	TSG	c.2956_2971del	p.P986Tfs*10	frameshift_variant	−/−
ZJUCH_19	Female	Neuroblastoma	*EXT2*	TSG	c.2005C>T	p.H669Y	missense_variant	−/−
ZJUCH_28	Male	Rhabdomyosarcoma	*NF1*	TSG	c.4103T>G	p.L1368*	stop_gained	−/−
ZJUCH_32	Female	Neuroblastoma	*APC*	TSG	c.1972_1975del	p.E658Tfs*11	frameshift_variant	−/−
ZJUCH_33	Female	Soft‐Tissue Sarcomas	*NBN*	TSG	c.1029dup	p.Q344Tfs*6	frameshift_variant	−/−
ZJUCH_46	Female	Nephroblastoma	*VHL*	TSG	c.341G>A	p.G114D	missense_variant	−/4
ZJUCH_48	Female	Neuroblastoma	*VHL*	TSG	c.607C>T	p.Q203*	stop_gained	−/−
ZJUCH_56	Female	Ganglioneuroma	*SDHB*	TSG	c.423+1G>A	/	splice_variant	−/−
ZJUCH_57	Male	Neuroblastoma	*RECQL4*	TSG	c.3072_3073del	p.V1026Afs*6	frameshift_variant	−/1
ZJUCH_59	Male	Hepatoblastoma	*SDHD*	TSG	c.341A>G	p.Y114C	missense_variant	−/−
ZJUCH_60	Female	Nephroblastoma	*AR*	Oncogene	c.1208C>T	p.A403V	missense_variant	−/3
ZJUCH_74	Male	Neurofibroma	*FANCA*	TSG	/	/	Deletion	−/−
ZJUCH_85	Male	Rhabdomyosarcoma	*TP53*	TSG	c.818G>A	p.R273H	missense_variant	251/1404
ZJUCH_90	Female	Neuroblastoma	*NF1*	TSG	c.730 + 1G>C	/	splice_variant	−/−
ZJUCH_92	Male	Nerve Sheath Tumor	*NF1*	TSG	c.2903T>G	p.M968R	missense_variant	−/−
ZJUCH_93	Female	Adrenocortical carcinoma	*TP53*	TSG	c.797G>A	p.G266E	missense_variant	35/152

*Note:* “−/−” indicates that no recurrence of identical amino acid changes was found in the corresponding database, with the result before the slash representing the query from the Cancer Hotspots database and the result after the slash representing the query from the COSMIC Cancer Mutation Census (CMC) database.

Tumor subtype‐specific analysis revealed: 6 neuroblastomas (26.1%, 6/23) with variants in *APC*, *EXT2*, *NF1*, *RECQL4*, *SDHB*, and *VHL*; 6 sarcomas (11.3%, 6/53) involving *DICER1*, *NF1* (*n* = 2), *NBN*, *FANCA*, and *TP53* (Table 1). Intriguingly, blastoma subtypes (6 neuroblastomas, 2 nephroblastomas, 1 hepatoblastoma) demonstrated a higher germline variant detection rate (27.3%, 9/33 vs. 11.5%, 7/61 in non‐blastomas, *p* = 0.08 by Fisher's exact test), suggesting embryonic‐origin tumors may possess distinct genetic susceptibility profiles (Table 1, Figure S2).

Notably, among the 16 patients with germline P/LP variants, we identified second somatic hits in 4 cases (25%), including three (ZJUCH_28, ZJUCH_85, ZJUCH_93) with identical somatic mutations matching their germline variants and one (ZJUCH_13) with an additional distinct somatic pathogenic variant in the same gene (*TP53*), supporting biallelic inactivation consistent with Knudson's two‐hit hypothesis (Table 1, Table S3).

### 3.4 Somatic Variants in Pediatric Solid Tumors

We identified 60 pathogenic/likely pathogenic SNVs and InDels across 38 patients (Figure 2a, Table S3). *TP53* emerged as the most frequently mutated gene (*n* = 9), followed by *NF1* (*n* = 6), *CTNNB1* (*n* = 4), *ALK* (*n* = 3), and *FBXW7* (*n* = 3). Tumor subtype‐specific analysis revealed: in sarcomas, *TP53* (*n* = 6) and *NF1* (*n* = 4) showed the highest mutation frequencies; among blastomas, *ALK* showed the highest mutation frequencies (3 neuroblastomas), with subsequent *TP53* (*n* = 2) and *NF1* (*n* = 2) alterations. Notably, *TP53* mutations demonstrated pan‐cancer distribution, with 2 cases exhibiting double P/LP variants. Interestingly, patient ZJUCH_13 harbored concurrent *TP53* deletion and SNV at homologous chromosomes, resulting in complete functional loss (Figure S4). Four patients carried *CTNNB1* P/LP variants (2 fibrosarcomas, 1 hepatic tumor, 1 adrenocortical carcinoma), while 5 additional cases exhibited *CTNNB1* variants of uncertain significance (VUS) (3 hepatoblastomas, 1 hepatic tumor, 1 nephroblastoma) (Figure S5, Table S4). This mutational spectrum aligns with established associations between *CTNNB1* aberrations and hepatic malignancies/fibrosarcomas reported in the literature [21, 22].

### 3.5 Copy Number Variations in Pediatric Solid Tumors

To account for tumor heterogeneity and pre‐treatment effects on DNA integrity, we established copy number thresholds (loss: ≤ 1.3; gain: ≥ 3.8). CNVs were identified in 44 patients (46.8%), encompassing 91 genes (Figure 2b, Figure S6, Table S5). *MYCN* amplifications predominated (*n* = 9), with amplification (≥ 12 copies, range 16–71) observed in 30.4% (7/23) of neuroblastomas, a hallmark of high‐risk stratification [23, 24]. Other recurrently amplified genes included *CDK4* (*n* = 5), *MDM2* (*n* = 2), *CCND2* (*n* = 1), and *SOS1* (*n* = 1). *CDK4*, a pivotal cell cycle regulator, demonstrated oncogenic activation patterns consistent with pan‐cancer proliferation dysregulation. *FGFR1* gains (4–6 copies) were sarcoma‐specific (4 cases, including 3 RMS), with all FGFR family P/LP variants (*FGFR1*/*FGFR4*) restricted to RMS (29.2% vs. blastomas 0%, *p* = 0.009 by Fisher's exact test), indicating FGFR signaling specificity in RMS pathogenesis. Notably, *MYC* amplifications were sarcoma‐exclusive, whereas *MDM2* amplifications were neuroblastoma‐specific, highlighting tumor‐type molecular divergence.

Frequent deletions involved *TP53* (*n* = 7), *SMARCB1* (*n* = 6), *CDKN2A* (*n* = 5), and *TNFRSF14* (*n* = 5). *SMARCB1* deletions exhibited sarcoma specificity, aligning with its diagnostic utility in sarcomagenesis (Figure 2b). Co‐deletions of SWI/SNF complex members *SMARCB1*/*SMARCA4* occurred in 2 sarcomas, with additional *SMARCA4* deletions in 2 neuroblastomas. Cell cycle deregulation was evident in 36.4% (16/44) of CNV‐positive cases, involving oncogenes (*CDK4* (*n* = 5), *CCND1/2* (*n* = 3), *CCNE1* (*n* = 1)) and tumor suppressors (*CDKN2A/2C* (*n* = 7), *CDKN1B* (*n* = 1)), underscoring their central oncogenic role (Figure S6).

Chromosomal CNVs hotspots included 1p (*n* = 25), 17q (*n* = 15), and 22q (*n* = 13). Compared to RMS and Non‐RMS, neuroblastomas showed significant 1p (16 vs. 7, *p* = 0.02 by Fisher's exact test) and 2p (9 vs. 1, *p* = 0.001 by Fisher's exact test) CNVs enrichment (Figures S7 and S8). Sarcoma‐specific 22q alterations and X chromosome abnormalities in Non‐RMS sarcomas (12 vs. 2, *p* < 0.001 by Fisher's exact test) revealed tumor‐type genomic vulnerabilities (Figure S9).

### 3.6 Chromosome Arm Aneuploidies in Pediatric Solid Tumors

**FIGURE 3:**
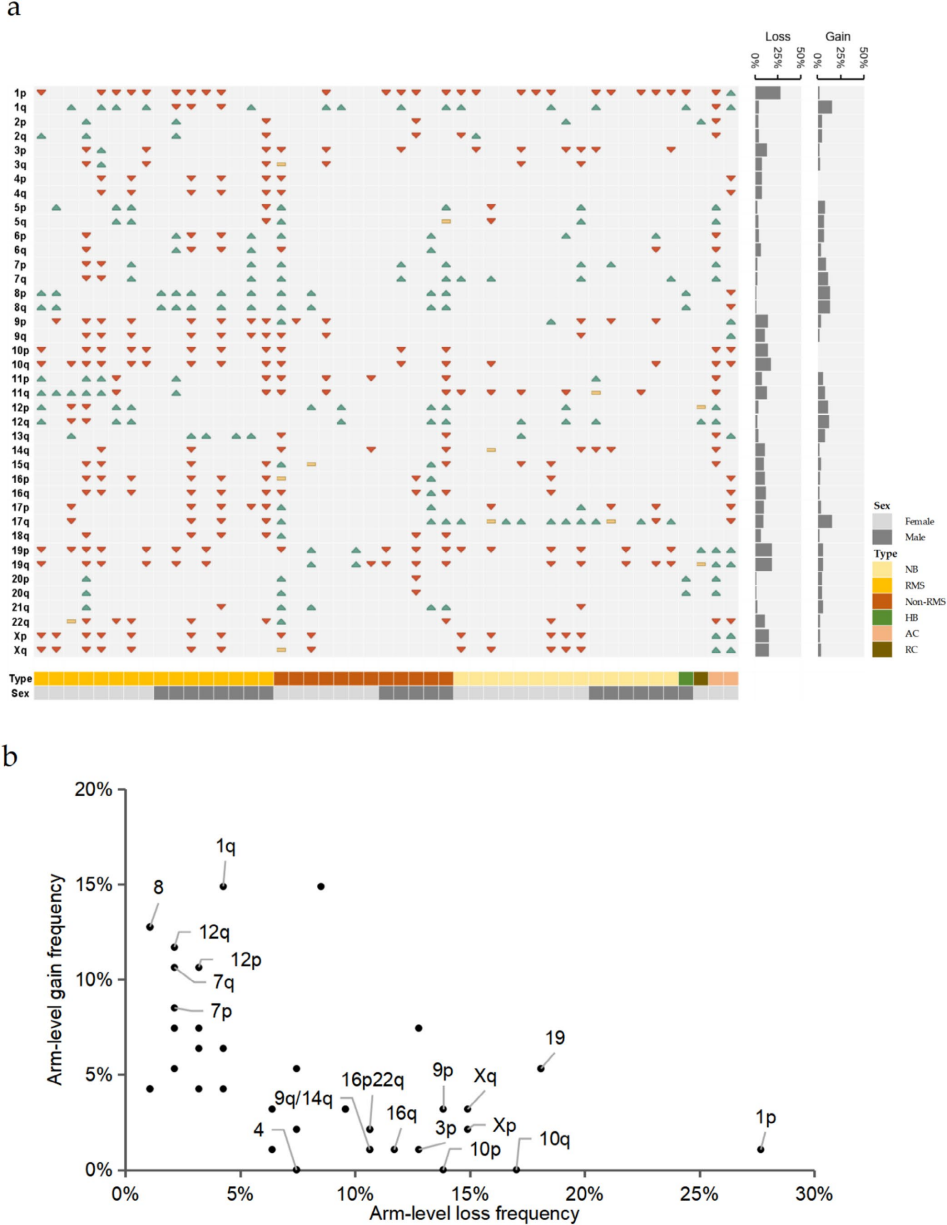
Loss events significantly outweighs gain events in samples with chromosomal arm aneuploidy. (a) Chromosome arm aneuploidies in the cohort. Red downward—pointing triangles indicate loss, green upward—pointing triangles indicate gain, and yellow rectangles indicate that both loss and gain are present on this arm. The panel on the right shows the frequency of loss or gain events on the chromosomal arms. Tumor type and sex are represented across the bottom. AC, adrenocortical carcinoma; HB, hepatoblastoma; NB, neuroblastoma; non‐RMS, non‐rhabdomyosarcoma; RC, renal cell carcinoma; RMS, rhabdomyosarcoma; (b) frequency of loss and gain on chromosomal arms. A binomial statistical analysis was performed on loss and gain events on the same chromosomal arm, and the names of points with *p*‐values < 0.05 were marked.

Leveraging the genomic coverage of our targeted sequencing panel, we detected chromosome arm aneuploidies (CAAs) by analyzing coordinated copy number alterations across gene clusters on chromosomal arms (Figure S10). Due to probe design limitations, 13p, 14p, 15p, 18p, 21p, 22p, and chromosome Y were excluded from analysis. Recurrent chromosomal arm deletions clustered on 1p (*n* = 26), 19 (*n* = 17), and 10q (*n* = 16), while predominant gains occurred at 1q (*n* = 14), 17q (*n* = 14), 8 (*n* = 12), and 12 (*n* = 10) (Figure 3a, Table S6).

Tumor subtype‐specific patterns emerged: 1p deletions and 1q gains were ubiquitous across neuroblastoma (NB), rhabdomyosarcoma (RMS), and non‐RMS sarcomas, whereas chromosome 8 gains were sarcoma‐specific (Figure S11). RMS exhibited significantly higher 9/10 chromosome deletion frequencies compared to NB (*p* = 0.02 by Fisher's exact test) and Non‐RMS (*p* = 0.03 by Fisher's exact test). Notably, 17q gains demonstrated NB predominance over RMS (*p* < 0.001 by Fisher's exact test) and Non‐RMS (*p* = 0.004 by Fisher's exact test).

Genome‐wide analysis revealed copy number losses (*n* = 309) significantly outnumbered gains (*n* = 189, *p* < 0.001 by Binomial statistics), with distinct chromosomal arm preferences: 1q, 7, 8, 12 showed gain propensity versus 1p, 3p, 4, 9, 10, 14q, 16, 19, 22p, and X with loss bias (Figure 3b). NB displayed borderline significant 17q gain enrichment (*p* = 0.053 by Fisher's exact test) (Figure S12). Sarcoma subtype stratification identified RMS‐specific 8/13q gains and Non‐RMS preferential gains at 1q, 8, 12p, and 21q (*p* = 0.063 by Fisher's exact test). RMS carried significantly higher deletion burdens than NB (15 vs. 7 arms, *p* = 0.08 by Fisher's exact test) and Non‐RMS (15 vs. 2 arms, *p* < 0.001 by Fisher's exact test).

**FIGURE 4:**
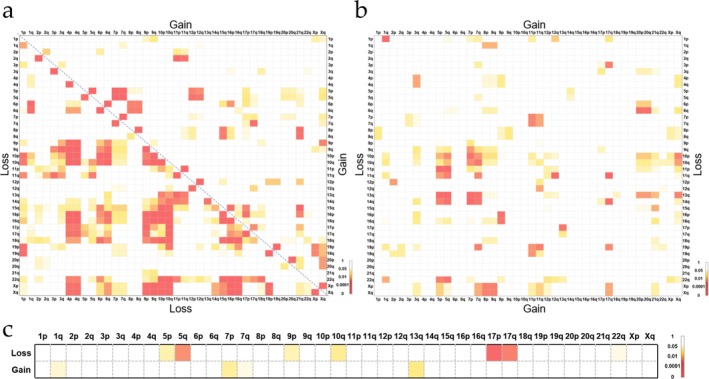
Co‐occurrence of chromosomal arm aneuploidy in pediatric solid tumors. (a) Fisher's exact test was used to calculate the co‐occurrence probability of loss—loss and gain—gain events on the same chromosomal arm. The area below the dashed line on the left represents loss—loss, while the area above the dashed line on the right represents gain—gain. (b) Fisher's exact test was used to calculate the co‐occurrence probability of loss—gain events on the same chromosomal arm. (c) Fisher's exact test was used to calculate the co‐occurrence probability of TP53 alterations and chromosomal arm aneuploidy. Areas filled with color represent *p*‐values < 0.05, and the darker the color, the smaller the *p‐*value.

Chromosomal co‐alterations, such as the diagnostic 1p/19q co‐deletion in oligodendrogliomas, have been well documented in oncogenesis [25]. Our study identified significant chromosomal co‐alteration patterns, with loss‐loss arm combinations occurring more frequently than gain‐gain or loss‐gain pairs (Figure 4a,b).

Neuroblastomas (NB) demonstrated limited aneuploidy co‐occurrence, primarily featuring 19p loss—7q gain, 11q loss—17q gain, and 19p—X co‐deletions (Figure S13). In contrast, rhabdomyosarcomas (RMS) and non‐RMS sarcomas exhibited higher co‐occurrence frequencies: RMS predominated by loss‐loss pairs (e.g., 4p/4q—9p/9q/10p/10q/16p/16q), whereas Non‐RMS showed gain‐gain preference (e.g., 8p/8q—12p/15q/17q/21q). Strikingly, *TP53* alterations were strongly associated with aneuploidy (14/47 cases, 29.8%; *p* < 0.001 by Fisher's exact test), demonstrating specific co‐occurrence with 5p/5q/9p/10q/17p/17q/22q losses and 1q/7p/13q gains (Figure 4c, Table S6).

### 3.7 Clinically Actionable Variants in Pediatric Solid Tumors

**FIGURE 5:**
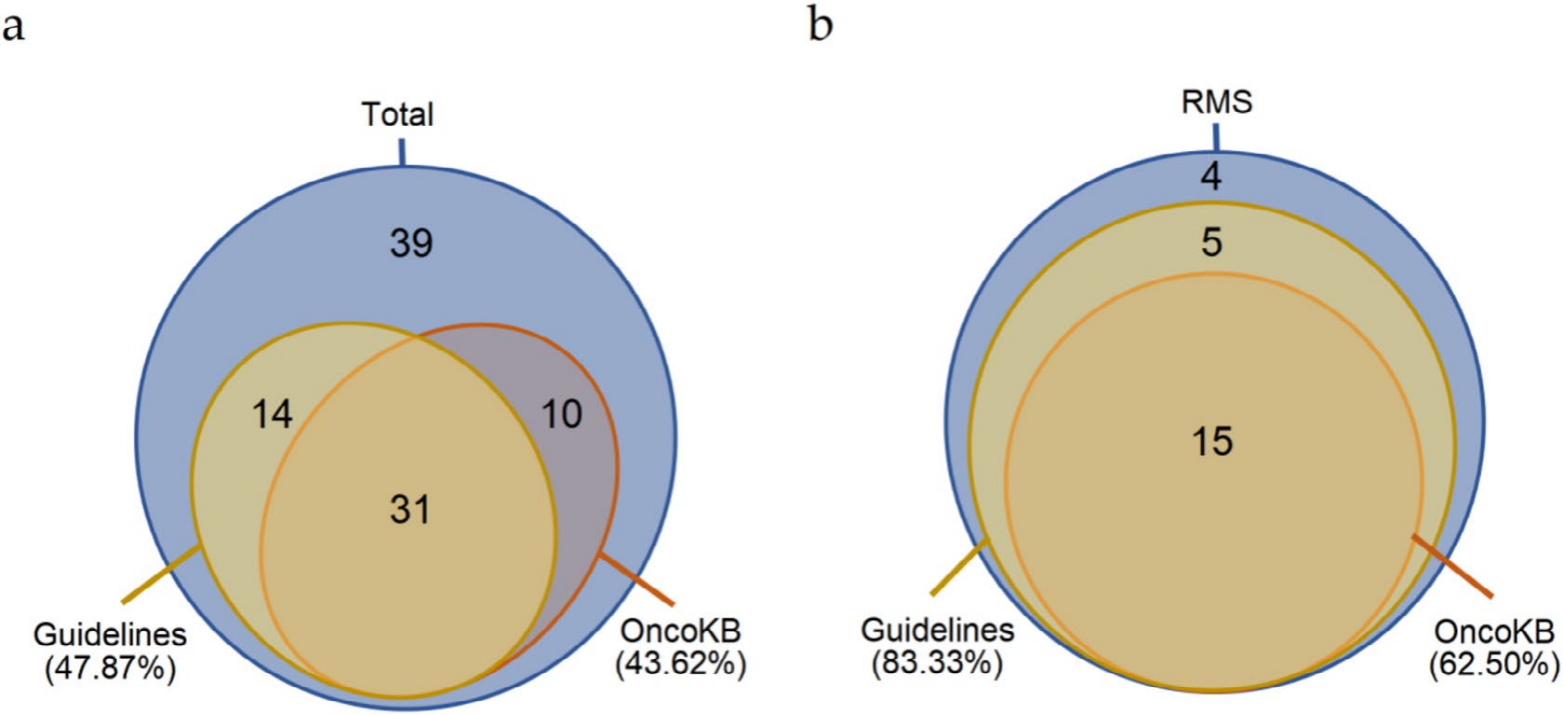
Clinically actionable variants in (a) all tumors and (b) rhabdomyosarcomas. “Guidelines” indicates that the gene is included in the NCCN guidelines for the corresponding tumor type, while “OncoKB” indicates that the mutation site can be matched with targeted therapy information in the OncoKB database. The numbers in the figure represent the number of cases that match this area, with the proportion of cases detected in this category shown in parentheses.

Clinically actionable variants (see Section 2.4) were identified in 58.5% (55/94) of patients, with 43.6% (41/94) harboring targeted therapeutic implications (Figure 5a). Notably, 27.7% (26/94) of these variants were classified as Level 1/R1 evidence (strong clinical evidence) according to OncoKB criteria. Eight patients demonstrated *NF1* alterations with therapeutic relevance. Guideline‐recommended genes (see Section 2.4) were detected in 47.9% (45/94) of cases, including sarcoma‐associated *SMARCB1/CDKN2A* deletions and *CDK4* amplifications, as well as *ALK* P/LP variants and *MYCN* amplifications in neuroblastomas. Strikingly, rhabdomyosarcomas exhibited a significantly higher prevalence of clinically actionable findings (83.3%, 20/24 vs. other subtypes, *p* = 0.004 by Fisher's exact test) (Figure 5b, Figure S14). These pieces of information provide molecular rationale for targeted therapeutic strategies.

## 4 Discussion

This study systematically characterized genomic profiles of 94 Chinese pediatric solid tumor cases using targeted NGS, delineating germline predisposition variants, somatic SNVs, InDels, and CNVs. Integrative genomic analysis revealed histology‐specific chromosomal instability patterns, particularly highlighting distinct chromosomal arm aneuploidy profiles between neuroblastomas and sarcomas, thereby providing novel insights into the genomic architecture of pediatric solid malignancies.

Pediatric malignancies exhibit distinct pathogenic mechanisms compared to adult cancers [4, 5]. Their development predominantly originates from malignant transformation of undifferentiated primordial cells during embryogenesis, contrasting with adult tumors driven primarily by environmentally induced somatic mutation accumulation, where genetic predisposition plays a more prominent role [3, 26, 27]. The revised WHO classification system, integrating molecular pathology features, has restructured pediatric tumor taxonomy, emphasizing the critical role of genomic profiling in precision diagnosis and therapeutic decision‐making [10, 28].

Genomic investigations reveal unique genetic characteristics of childhood cancers: significantly elevated germline P/LP variant rates compared to adult malignancies, coupled with markedly lower somatic mutation burdens [3, 11, 26]. Multicenter cohort studies demonstrate: MSK‐IMPACT (*n* = 751) reported 18% P/LP variants [19]; whole‐genome/exome sequencing of 1120 patients by Zhang et al. [29] identified 8.5% cancer predisposition gene carriers with *TP53* predominance (50%); G4K and Chinese solid tumor cohorts (Jie Gong et al. [30]) showed 18% and 8.5% detection rates respectively [13, 30]. Our study revealed 17% P/LP variant prevalence, with recurrent alterations in *NF1* (*n* = 3), *TP53* (*n* = 2), and *VHL* (*n* = 2). The *NF1/TP53* mutation spectrum aligns with Chinese cohort findings, while *VHL* absence likely reflects tumor subtype heterogeneity [30].

Clinically, germline testing holds vital implications despite limited therapeutic impact: (1) ~40% of P/LP carriers lack familial cancer history [31], necessitating universal screening; (2) Definitive mutation identification enables family genetic counseling and early surveillance [32, 33]; (3) DNA repair defects (e.g., *TP53*, *BRCA2*) confer elevated secondary malignancy risks requiring personalized monitoring [31, 34]. These findings underscore the imperative to integrate germline analysis into standard pediatric oncology workflows [8, 11, 35].

Chromosomal aneuploidy, a hallmark genomic feature of malignancies, is prevalent in ~90% of solid tumors and 50% of hematopoietic cancers [36, 37, 38]. Recent pan‐cancer analyses demonstrate that clonal chromosome arm alterations (CAAs), involving whole‐arm or entire‐chromosome copy number changes, emerge during early tumorigenesis. Notably, hematologic and solid malignancies exhibit divergent aneuploidy evolutionary trajectories: both initially favor arm‐level gains, but only solid tumors progress to multi‐arm losses, aligning with our observed predominance of chromosomal loss events [39].

CAAs demonstrate superior predictive value in precision oncology compared to somatic mutations and focal CNVs [39]. Multivariate analysis of 72 CAAs and 88 synergistic CAA pairs predicted survival outcomes (favorable/adverse) in 58% of 6977 patients [39]. Mechanistically, chromosome instability (CIN)‐metastasis burden correlations show tissue‐specific patterns: strong positive associations in prostate adenocarcinoma, lung adenocarcinoma, and HR+/HER2+ breast cancer versus nonsignificant links in colorectal and high‐grade serous ovarian carcinomas, highlighting CAAs' dual prognostic and therapeutic predictive utility [40]. Our study uncovered subtype‐specific aneuploidy signatures in pediatric solid tumors: sarcoma‐characteristic chromosome 8 gains and neuroblastoma‐enriched 17q amplifications, markedly differing from adult patterns. Importantly, aneuploidy in neuroblastoma has been associated with a favorable prognosis, suggesting the prognostic potential of CAAs [41, 42, 43]. However, clinical translation of this finding requires validation in larger cohorts.

Vishruth Girish et al. [15] employed CRISPR‐mediated ReDACT technology to engineer isogenic cells that have or lack common aneuploidies, proving 1q trisomy essential for malignant progression in specific cancers. Intriguingly, while their study revealed mutual exclusivity between *TP53* mutations and 1q gains in adult tumors, we observed co‐occurrence in pediatric solid malignancies, suggesting distinct genomic regulatory networks in childhood cancers.

Nonetheless, these findings should be interpreted with caution, and several limitations warrant consideration. The study's statistical power was constrained by the inherent challenges of pediatric oncology recruitment and the lower incidence of childhood malignancies compared to adult cancers. Our cohort, predominantly composed of sarcomas and neuroblastomas, may not fully represent the molecular heterogeneity of pediatric solid tumors. Future investigations should prioritize expanding cohort diversity through multicenter collaborations, coupled with integrated multi‐omics approaches incorporating transcriptomic profiling and DNA methylation analysis. Such efforts will enable comprehensive mapping of pediatric tumor genomic landscapes, ultimately informing biomarker‐driven therapeutic strategies.

## Author Contributions

**Bingxiao Yan:** conceptualization (equal), methodology (equal), software (equal), Data curation (equal), Investigation (equal), formal analysis (equal), visualization (equal), writing – original draft. **Jinhu Wang:** conceptualization (equal), methodology (equal), validation (equal), funding acquisition (equal). **Jieni Xiong:** formal analysis (equal), investigation (equal), data curation (equal), visualization (equal). **Shuangai Liu:** investigation (equal). **Yinbing Tang:** resources (equal). **Ziqi He:** resources (equal). **Hujin Yan:** resources (equal). **Bize Guo:** resources (equal). **Chen Chen:** resources (equal). **Yijie Zhang:** resources (equal). **Qinfang Zhu:** resources (equal). **Jiabin Cai:** resources (equal). **Min He:** resources (equal). **Xuan Wu:** resources (equal). **Junqing Mao:** resources (equal). **Lifeng Zhang:** resources (equal). **Weizhong Gu:** project administration. **Zhu Zhu:** data curation (equal). **Zheming Li:** data curation (equal). **Rui Xiao:** methodology (equal), software (equal), validation (equal), supervision. **Qiang Shu:** conceptualization (equal), validation (equal). **Gang Yu:** conceptualization (equal), validation (equal). **Ting Tao:** conceptualization (equal), methodology (equal), validation (equal), writing – review and editing, funding acquisition (equal).

## Funding

This work was supported by the Key R&D Program of Zhejiang Province, No. 2024C03181, No. 2025C01106, National Natural Science Foundation of China, No. 32270853, No. U20A20137, Cancer Center, Zhejiang University, No. 20200108.

## Ethics Statement

The study was conducted in accordance with the Declaration of Helsinki and approved by the Ethics Committee of Children's Hospital, Zhejiang University School of Medicine (2020‐IRB‐049 and 2024‐IRB‐0191‐P‐01). All data were acquired with informed consent from patients, their parents or guardians. All results were reported with arbitrary sample ID numbers without linked identifiers.

## Conflicts of Interest

The authors declare no conflicts of interest.

## Supporting information


**Figure S1:** Number of cases with different variant types detected in (a) neuroblastomas, (b) rhabdomyosarcomas or (c) non‐rhabdomyosarcomas.
**Figure S2:** Germline and Somatic variants in tumors. (a) Germline and Somatic variants in total tumors. (b) Germline and Somatic variants in neuroblastomas. (c) Germline and Somatic variants in rhabdomyosarcomas. (d) Germline and Somatic variants in non‐rhabdomyosarcomas.
**Figure S3:** The list of germline predisposition genes varies across different projects. G4K is derived from “Genomes for Kids” [13], MSK‐IMPACT is derived from [15], Cosimc_CGC is derived from COSMIC Cancer Gene Census (CGC) database.
**Figure S4:** Patient ZJUCH_13 harbored concurrent *TP53* deletion and SNV at homologous chromosomes. IGV displays exon 7 and exon 8 of *TP53*, with red arrows indicating reads with SNV but no deletions, and blue arrows pointing to reads with deletions but no SNV. The upper black box represents the results from the patient's peripheral blood, while the lower black box shows the results from the tissue sample.
**Figure S5:** Genes containing variant of uncertain significance (VUS) SNV/InDel sites. Case count for each category is shown in the same color as the legend.
**Figure S6:** All mutated genes containing copy number variant (CNV). Red represents gene copy number duplication (dup), while green indicates gene copy number deletions (del). The classification of gene copy number is detailed in the legend. The number of variants identified per gene is represented to the right. Tumor type and sex are represented across the bottom. AC = adrenocortical carcinoma, HB = hepatoblastoma, NB = neuroblastoma, non‐RMS = non‐rhabdomyosarcoma, NpB = nephroblastoma, RC = renal cell carcinoma, RMS = rhabdomyosarcoma.
**Figure S7:** The distribution of genes with CNV variations on chromosomes. Dots represent genes, and colored dots indicate recurrently CNV genes, with gray dots representing genes detected only once. Except for the gray points, points of the same color represent the same gene detected in different individuals. The numbers in parentheses below the gene names indicate the number of times the genes were detected. The *x*‐axis represents chromosomal positions, with gray vertical lines indicating centromere positions, and the *y*‐axis represents gene copy numbers.
**Figure S8:** The distribution of genes with CNV variations on chromosomes 1. Dots represent genes, and colored dots indicate recurrently CNV genes, with gray dots representing genes detected only once. Except for the gray points, points of the same color represent the same gene detected in different individuals. The numbers in parentheses below the gene names indicate the number of times the genes were detected. The x‐axis represents chromosomal 1 positions, with gray vertical lines indicating centromere positions, and the y‐axis represents gene copy numbers.
**Figure S9:** The total number of CNV—altered genes detected on each chromosomal arm. The total number comprises the number of different genes on the same chromosomal arm in a single patient and the number of different patients with the same CNV gene. NB = neuroblastoma, non‐RMS = non‐rhabdomyosarcoma, RMS = rhabdomyosarcoma.
**Figure S10:** The distribution of targeted panel probe capture regions on chromosomes. The *x*‐axis represents chromosomal positions, with gray vertical lines indicating centromere positions.
**Figure S11:** Chromosome arm aneuploidies in (a) neuroblastomas, (b) rhabdomyosarcomas and (c) non‐rhabdomyosarcomas. Red downward—pointing triangles indicate loss, green upward—pointing triangles indicate gain, and yellow rectangles indicate that both loss and gain are present on this arm. The panel on the right shows the frequency of loss or gain events on the chromosomal arms.
**Figure S12:** Frequency of loss and gain on chromosomal arms in (a) neuroblastomas, (b) rhabdomyosarcomas and (c) non‐rhabdomyosarcomas. A binomial statistical analysis was performed on loss and gain events on the same chromosomal arm, and the names of points with *p*—values less than 0.05 were marked.
**Figure S13:** Fisher's exact test was used to calculate the co—occurrence probability of loss—loss and gain—gain events on the same chromosomal arm in (a) neuroblastomas, (c) rhabdomyosarcomas and (e) non‐rhabdomyosarcomas. The area below the dashed line on the left represents loss—loss, while the area above the dashed line on the right represents gain—gain. Fisher's exact test was used to calculate the co—occurrence probability of loss—gain events on the same chromosomal arm in (b) neuroblastomas, (d) rhabdomyosarcomas, and (f) non‐rhabdomyosarcomas. Areas filled with color represent *p*‐values less than 0.05, and the darker the color, the smaller the *p*‐value.
**Figure S14:** Clinically actionable variants in (a) neuroblastomas and (b) non‐rhabdomyosarcomas. “Guidelines” indicates that the gene is included in the NCCN guidelines for the corresponding tumor type, while “OncoKB” indicates that the mutation site can be matched with targeted therapy information in the OncoKB database. The numbers in the figure represent the number of cases that match this area, with the proportion of cases detected in this category shown in parentheses.

**Table S1:** Patients information.
**Table S2:** The list of panel genes and candidate genes examined for germline variants.
**Table S3:** The information of P/LP SNV/InDel somatic variants.
**Table S4:** The information of VUS SNV/InDel somatic variants.
**Table S5:** The information of CNV somatic variants.
**Table S6:** The information of chromosome arm aneuploidies.

## Data Availability

The targeted gene panel sequencing data generated in this study were available from the Genome Sequence Archive [44] in National Genomics Data Center [45], China National Center for Bioinformation/Beijing Institute of Genomics, Chinese Academy of Sciences under accession number HRA010447 (https://ngdc.cncb.ac.cn/gsa‐human/).
