# Supplementary material for: Integrative Genomic Profiling of Pediatric Solid Tumors Reveals Clinically Relevant Variants and Chromosomal Arm Aneuploidies Signatures
Source: Cancer Med. 2026 Mar 3;15(3):e71666. doi: 10.1002/cam4.71666 (PMC12956543; doi:10.1002/cam4.71666)
Supplement: Supplementary file 1 — Figure S1: Number of cases with different variant types detected in (a) neuroblastomas, (b) rhabdomyosarcomas or (c) non‐rhabdomyosarcomas. Figure S2: Germline and Somatic variants in tumors. (a) Germline and Somatic variants in total tumors. (b) Germline and Somatic variants in neuroblastomas. (c) Germline and Somatic variants in rhabdomyosarcomas. (d) Germline and Somatic variants in non‐rhabdomyosarcomas. Figure S3: The list of germline predisposition genes varies across different projects. G4K is derived from “Genomes for Kids” [13], MSK‐IMPACT is derived from [15], Cosimc_CGC is derived from COSMIC Cancer Gene Census (CGC) database. Figure S4: Patient ZJUCH_13 harbored concurrent TP53 deletion and SNV at homologous chromosomes. IGV displays exon 7 and exon 8 of TP53, with red arrows indicating reads with SNV but no deletions, and blue arrows pointing to reads with deletions but no SNV. The upper black box represents the results from the patient's peripheral blood, while the lower black box shows the results from the tissue sample. Figure S5: Genes containing variant of uncertain significance (VUS) SNV/InDel sites. Case count for each category is shown in the same color as the legend. Figure S6: All mutated genes containing copy number variant (CNV). Red represents gene copy number duplication (dup), while green indicates gene copy number deletions (del). The classification of gene copy number is detailed in the legend. The number of variants identified per gene is represented to the right. Tumor type and sex are represented across the bottom. AC = adrenocortical carcinoma, HB = hepatoblastoma, NB = neuroblastoma, non‐RMS = non‐rhabdomyosarcoma, NpB = nephroblastoma, RC = renal cell carcinoma, RMS = rhabdomyosarcoma. Figure S7: The distribution of genes with CNV variations on chromosomes. Dots represent genes, and colored dots indicate recurrently CNV genes, with gray dots representing genes detected only once. Except for the gray points, points of the same colo [file CAM4-15-e71666-s002.docx]

a b


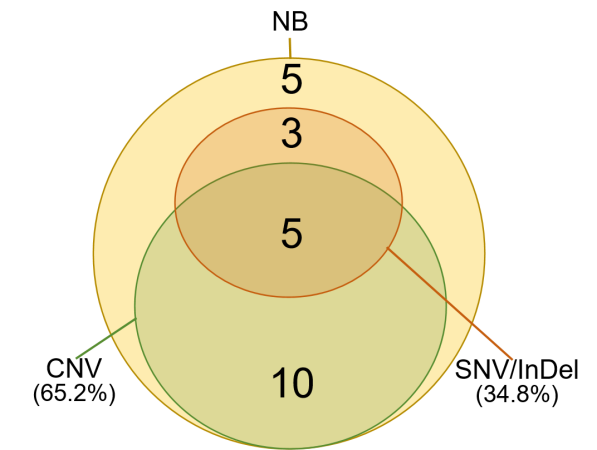

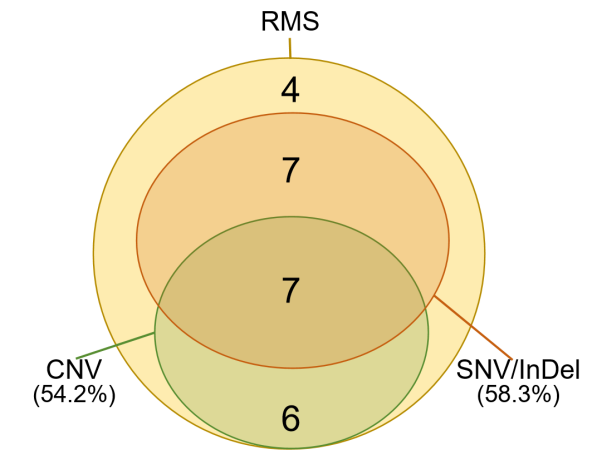


c
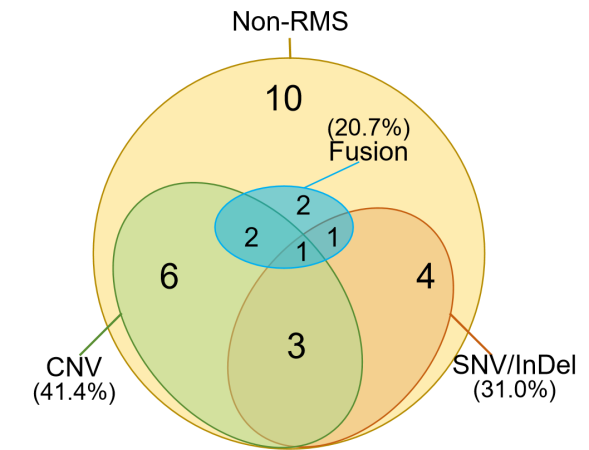


**Supplementary Figure S1.** Number of cases with different variant types detected in (a) neuroblastomas, (b) rhabdomyosarcomas or (c) non-rhabdomyosarcomas.

a b


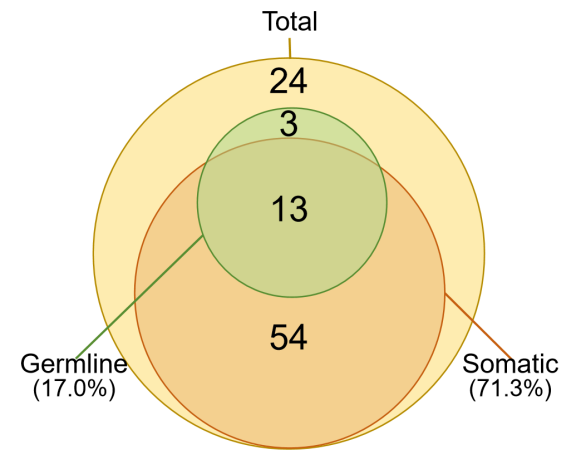

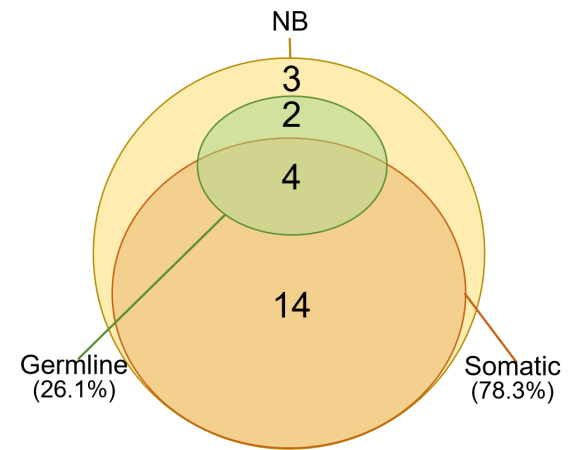


c d


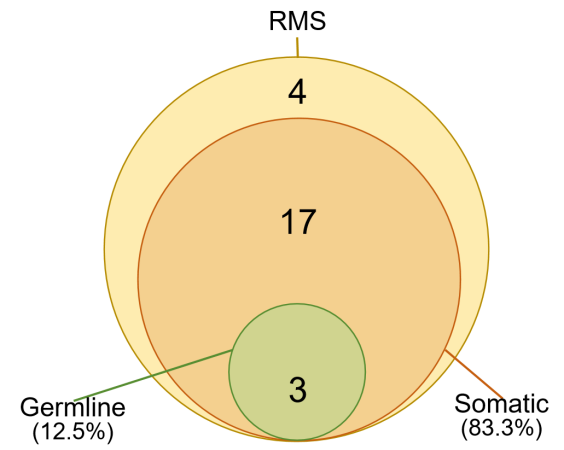

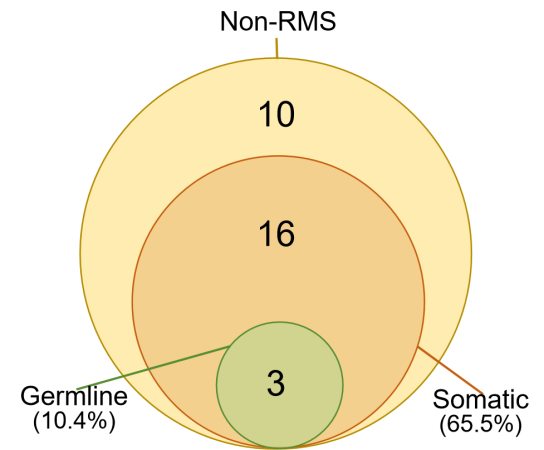


**Supplementary Figure S2.** Germline and Somatic variants in tumors. (a) Germline and Somatic variants in total tumors. (b) Germline and Somatic variants in neuroblastomas. (c) Germline and Somatic variants in rhabdomyosarcomas. (d) Germline and Somatic variants in non-rhabdomyosarcomas.


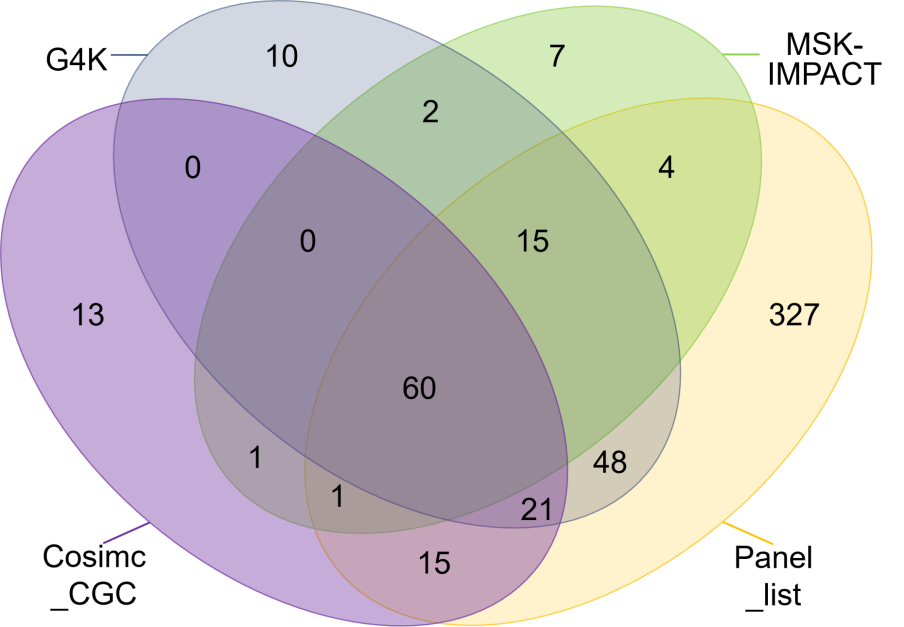


**Supplementary Figure S3.** The list of germline predisposition genes varies across different projects. G4K is derived from "Genomes for Kids"[13], MSK-IMPACT is derived from [15], Cosimc_CGC is derived from COSMIC Cancer Gene Census (CGC) database.


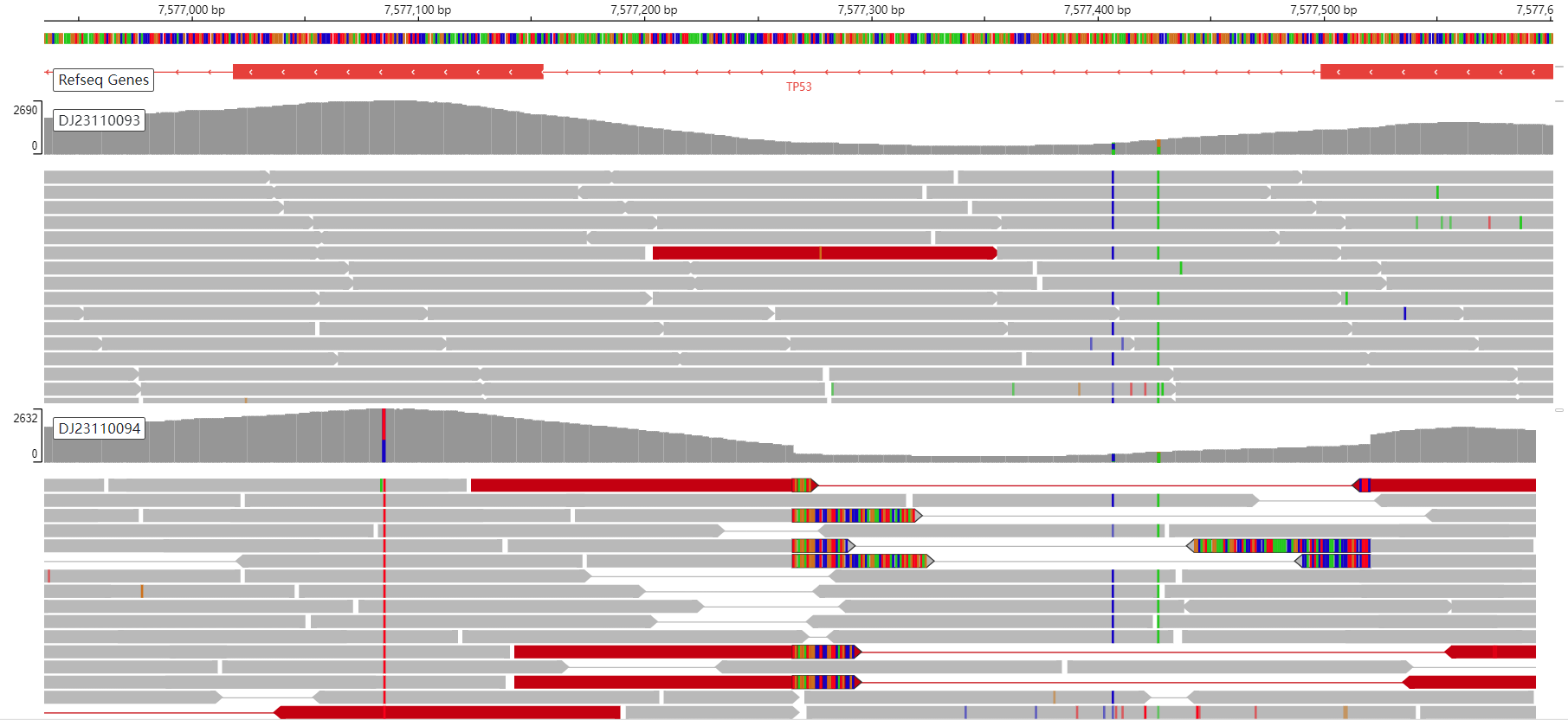

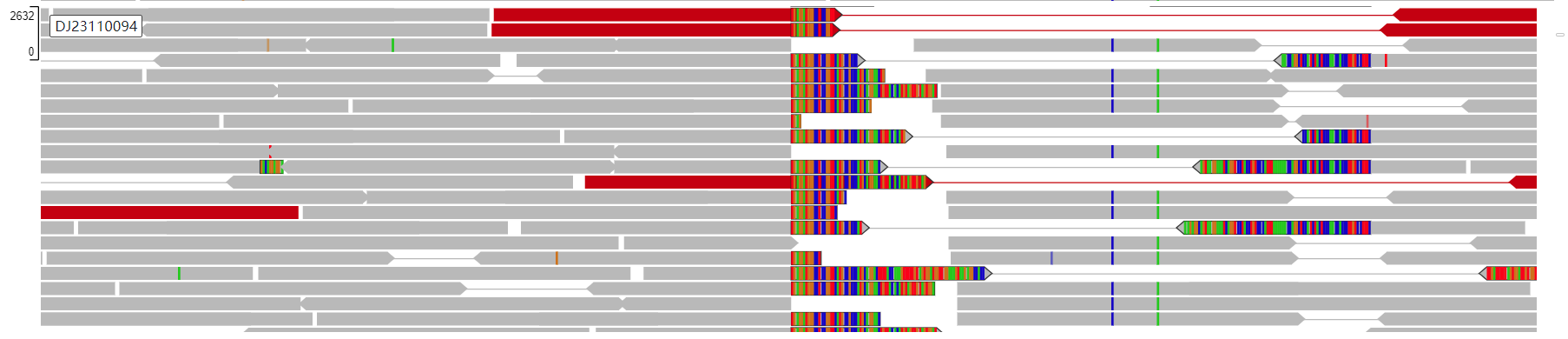


Exon8 Exon7

**Supplementary Figure S4.** Patient ZJUCH_13 harbored concurrent *TP53* deletion and SNV at homologous chromosomes. IGV displays exon 7 and exon 8 of *TP53*, with red arrows indicating reads with SNV but no deletions, and blue arrows pointing to reads with deletions but no SNV. The upper black box represents the results from the patient's peripheral blood, while the lower black box shows the results from the tissue sample.

**Supplementary Figure S5.** Genes containing variant of uncertain significance (VUS) SNV/InDel sites. Case count for each category is shown in the same colour as the legend.


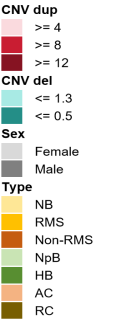


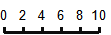


Patients of gene

**Supplementary Figure S6.** All mutated genes containing copy number variant (CNV). Red represents gene copy number duplication (dup), while green indicates gene copy number deletions (del). The classification of gene copy number is detailed in the legend. The number of variants identified per gene is represented to the right. Tumor type and sex are represented across the bottom. NB = neuroblastoma, RMS = rhabdomyosarcoma, Non-RMS = non-rhabdomyosarcoma, NpB = nephroblastoma, HB = hepatoblastoma, AC = adrenocortical carcinoma, RC = renal cell carcinoma.


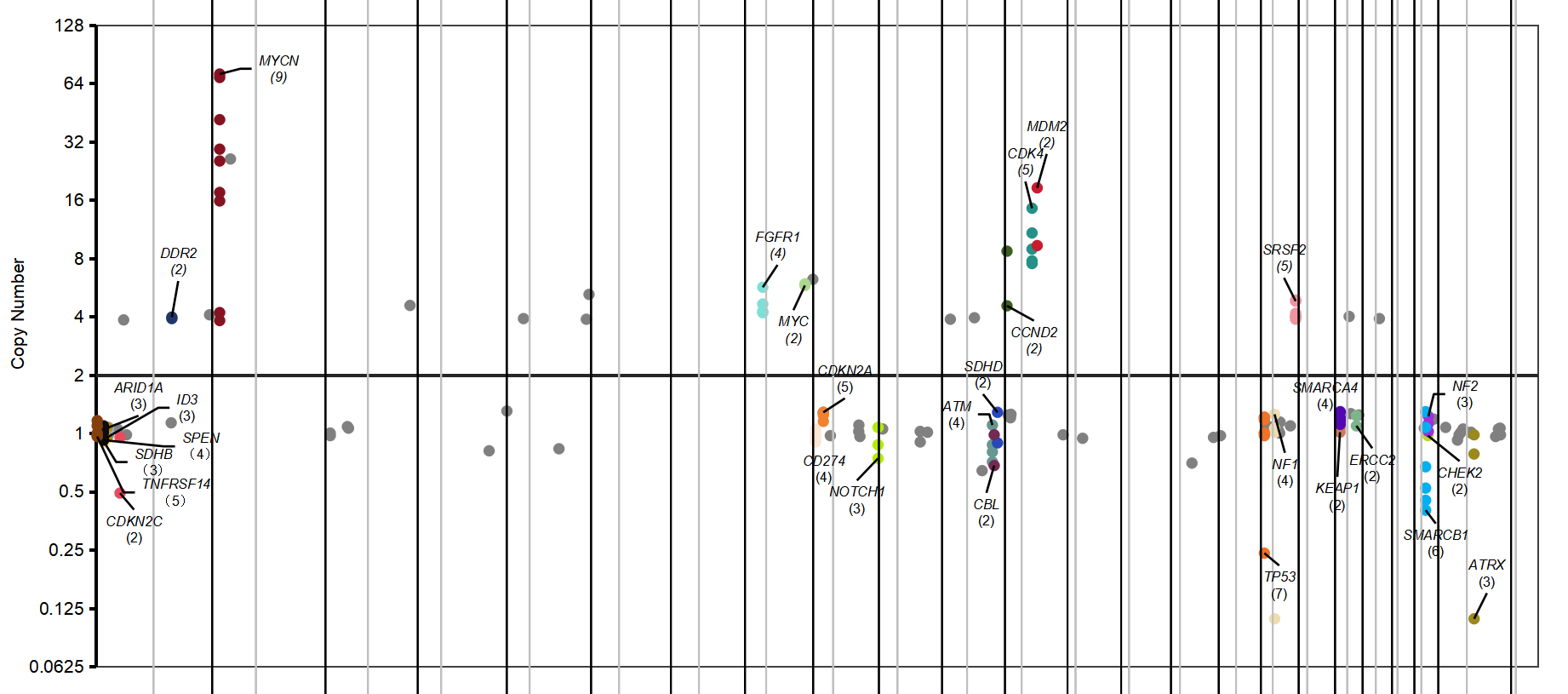


1 2 3 4 5 6 7 8 9 10 11 12 13 14 15 16 17 18 19 20 21 22 X Y

Chromosomes

Chromosomes

**Supplementary Figure S7.** The distribution of genes with CNV variations on chromosomes. Dots represent genes, and colored dots indicate recurrently CNV genes, with gray dots representing genes detected only once. Except for the gray points, points of the same color represent the same gene detected in different individuals. The numbers in parentheses below the gene names indicate the number of times the genes were detected. The x-axis represents chromosomal positions, with gray vertical lines indicating centromere positions, and the y-axis represents gene copy numbers.


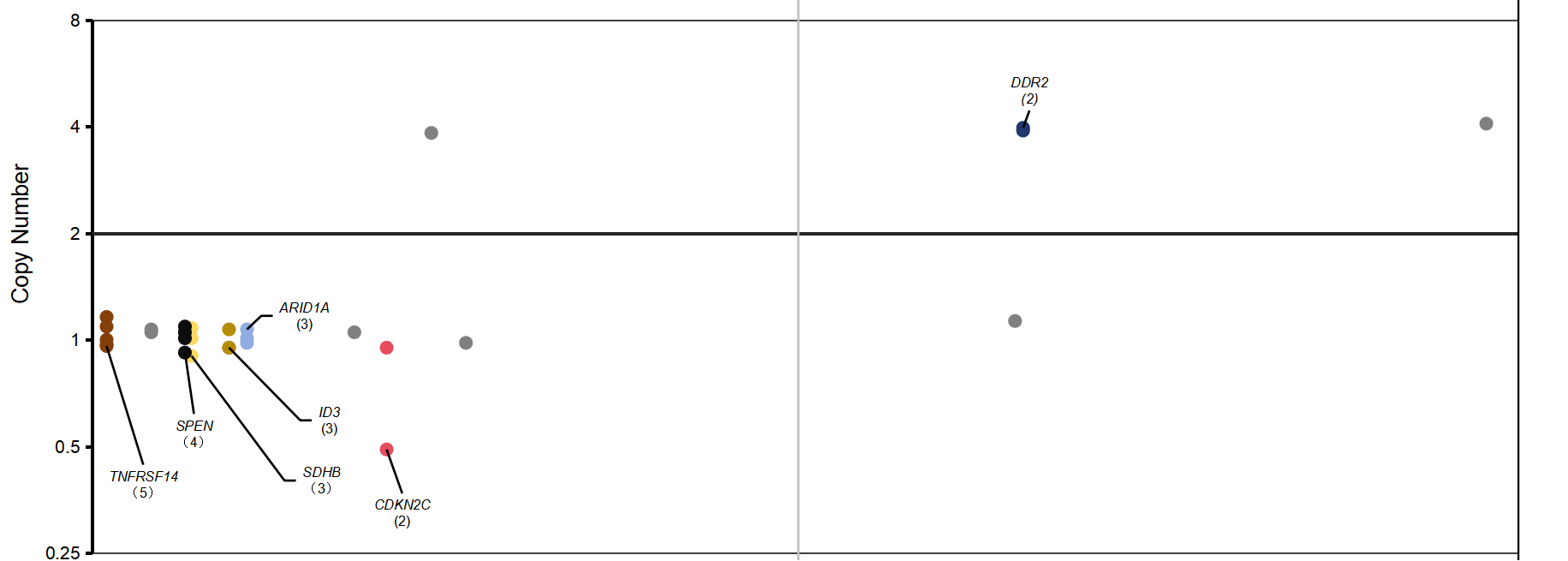

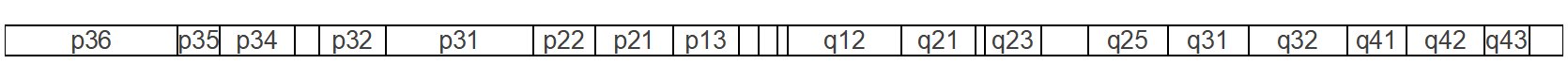


Chr1

**Supplementary Figure S8.** The distribution of genes with CNV variations on chromosomes 1. Dots represent genes, and colored dots indicate recurrently CNV genes, with gray dots representing genes detected only once. Except for the gray points, points of the same color represent the same gene detected in different individuals. The numbers in parentheses below the gene names indicate the number of times the genes were detected. The x-axis represents chromosomal 1 positions, with gray vertical lines indicating centromere positions, and the y-axis represents gene copy numbers.


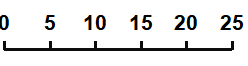


**Number of genes**


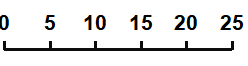


**Number of genes**


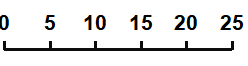


**Number of genes**


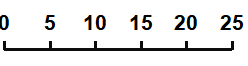


**Number of genes**

**Supplementary Figure S9.** The total number of CNV - altered genes detected on each chromosomal arm. The total number comprises the number of different genes on the same chromosomal arm in a single patient and the number of different patients with the same CNV gene. NB = neuroblastoma, RMS = rhabdomyosarcoma, Non-RMS = non-rhabdomyosarcoma.

**Supplementary Figure S10.** The distribution of targeted panel probe capture regions on chromosomes. The x-axis represents chromosomal positions, with gray vertical lines indicating centromere positions.


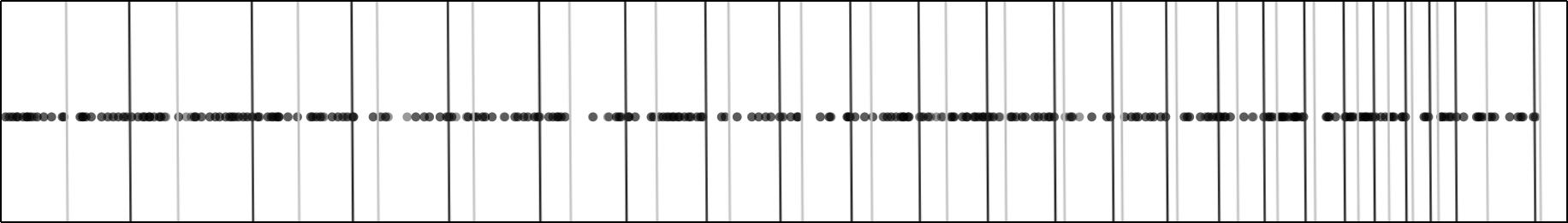


1 2 3 4 5 6 7 8 9 10 11 12 13 14 15 16 17 18 19 20 21 22 X Y Chromosomes

a b c


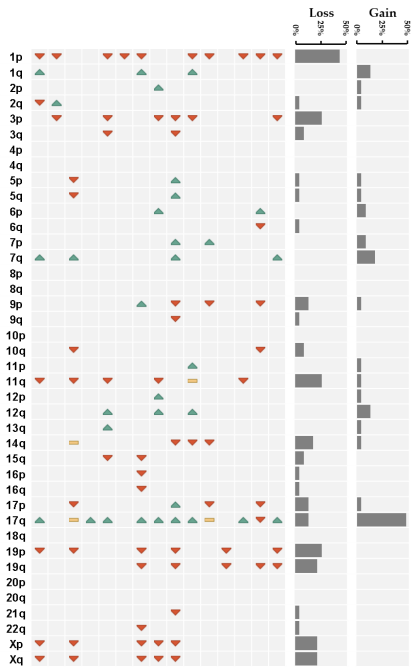

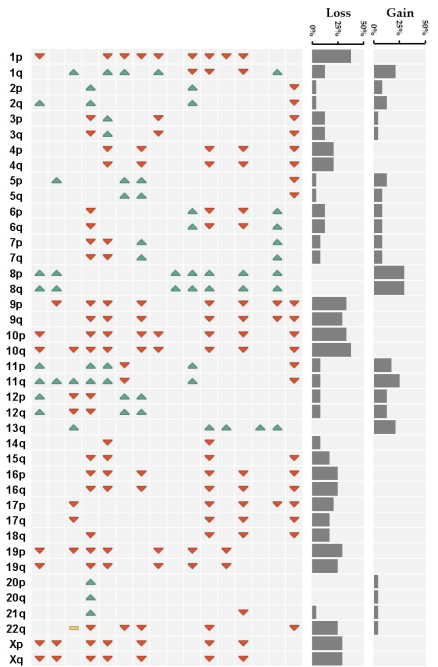

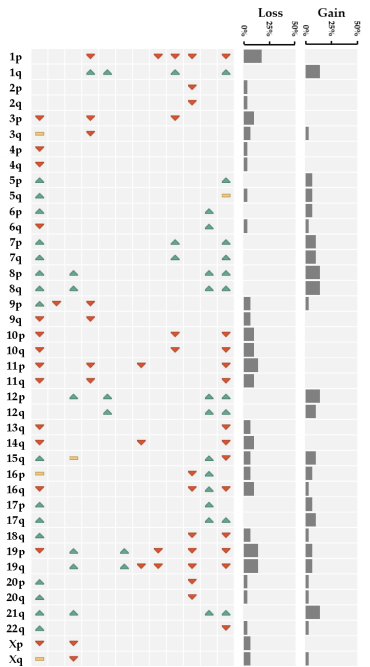


**Supplementary Figure S11.** Chromosome arm aneuploidies in (a) neuroblastomas, (b) rhabdomyosarcomas and (c) non-rhabdomyosarcomas. Red downward - pointing triangles indicate loss, green upward - pointing triangles indicate gain, and yellow rectangles indicate that both loss and gain are present on this arm. The panel on the right shows the frequency of loss or gain events on the chromosomal arms.

a b

c

**Supplementary Figure S12.** Frequency of loss and gain on chromosomal arms in (a) neuroblastomas, (b) rhabdomyosarcomas and (c) non-rhabdomyosarcomas. A binomial statistical analysis was performed on loss and gain events on the same chromosomal arm, and the names of points with p - values less than 0.05 were marked.

a b


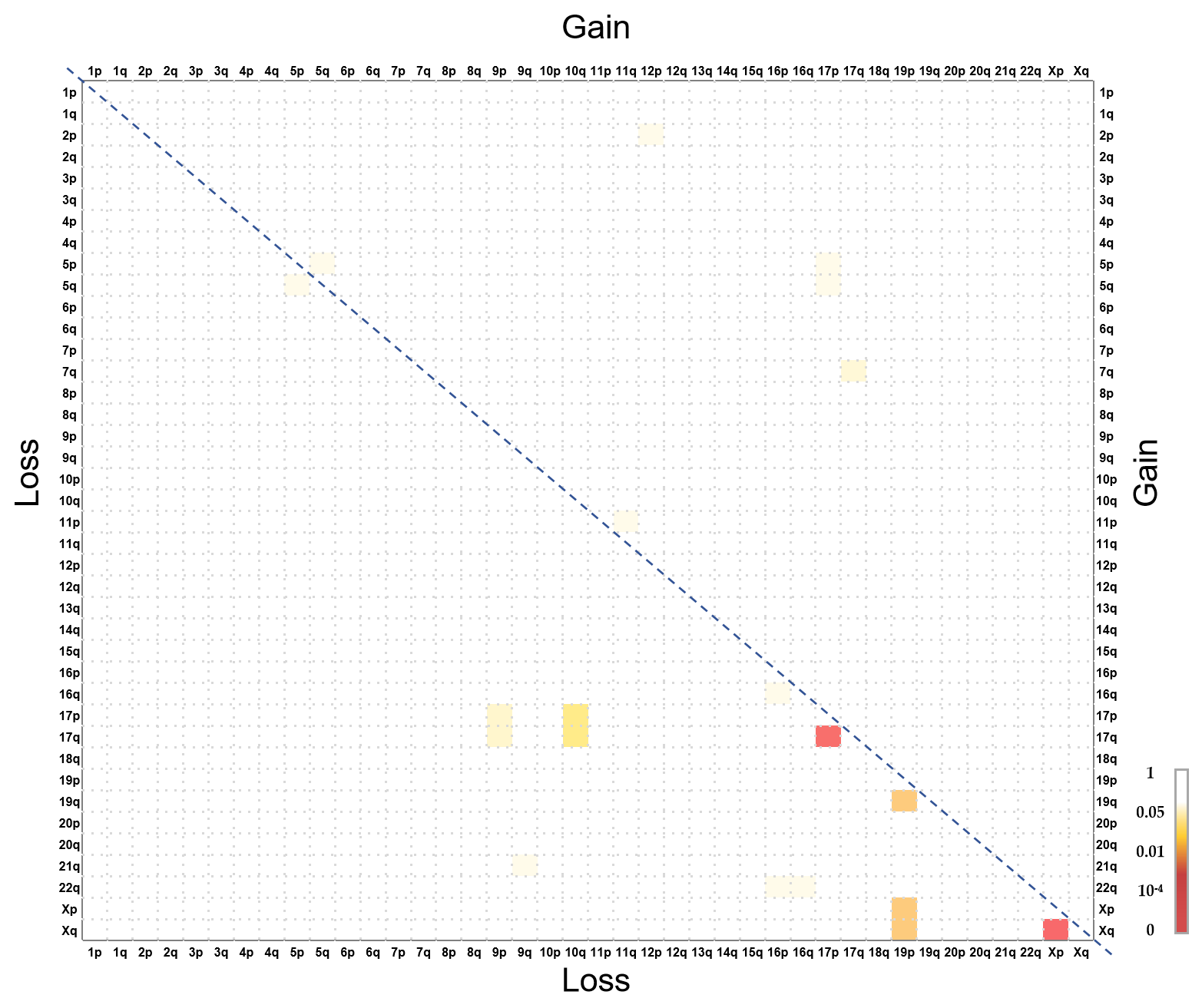

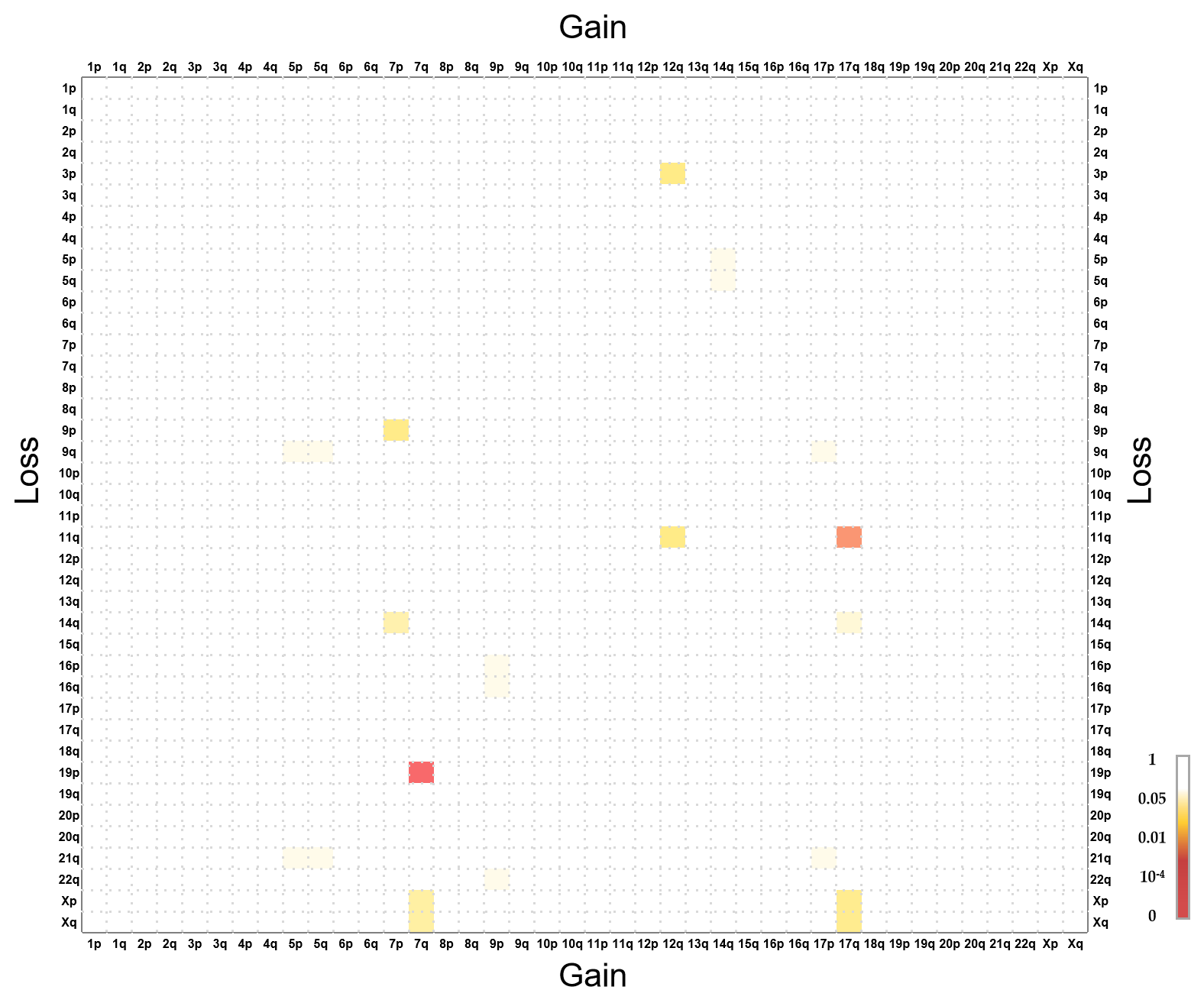


c d


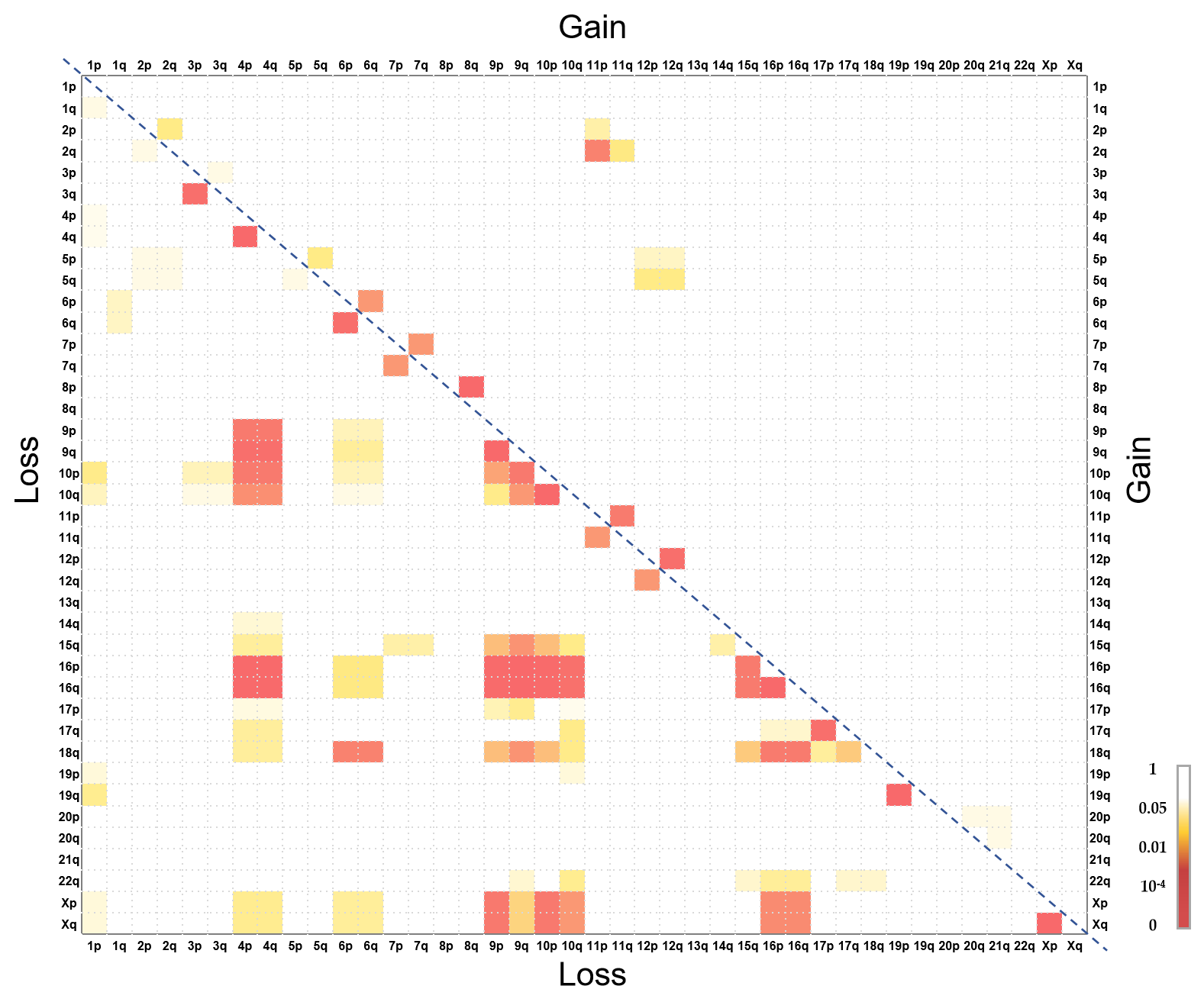

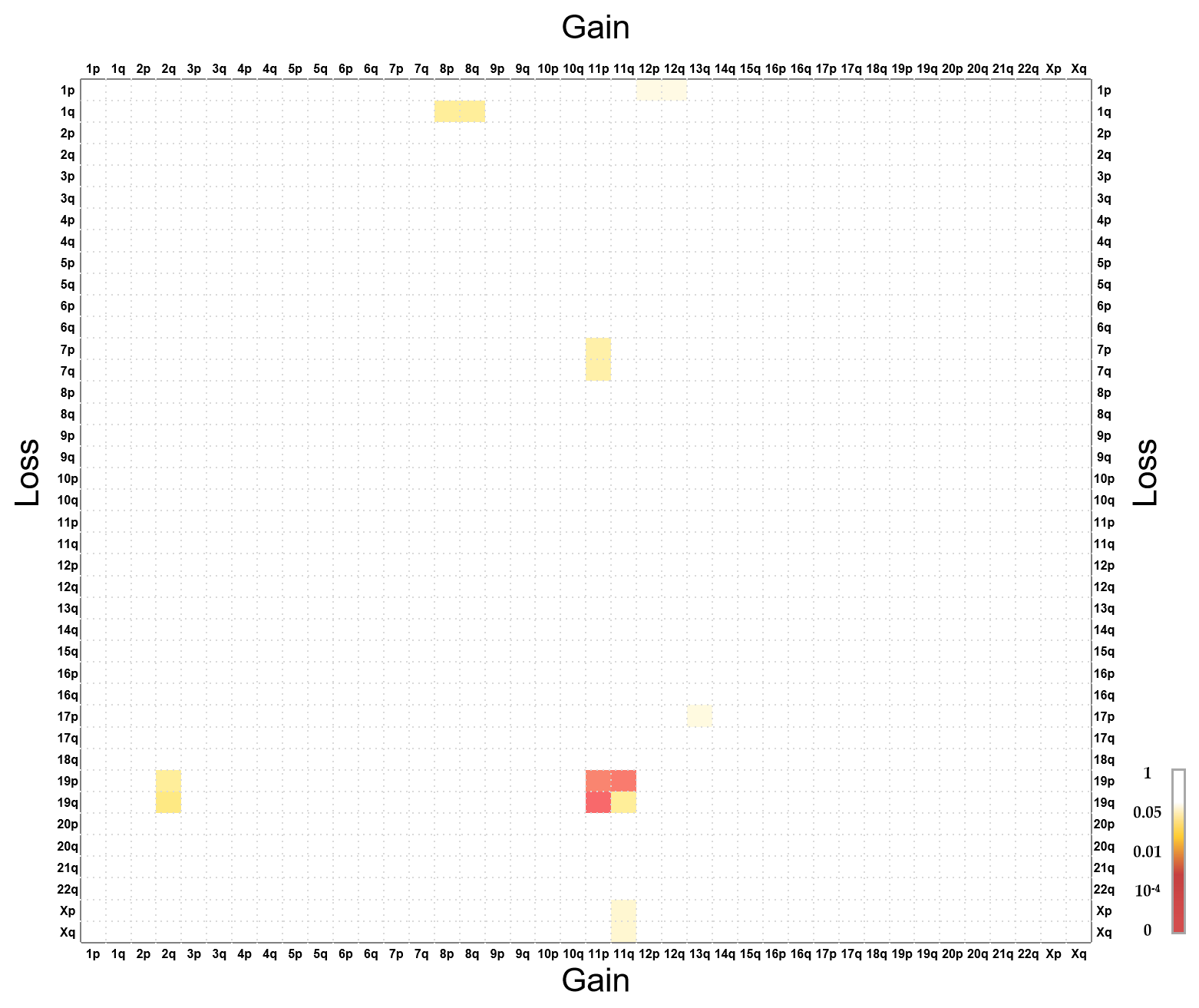


e f


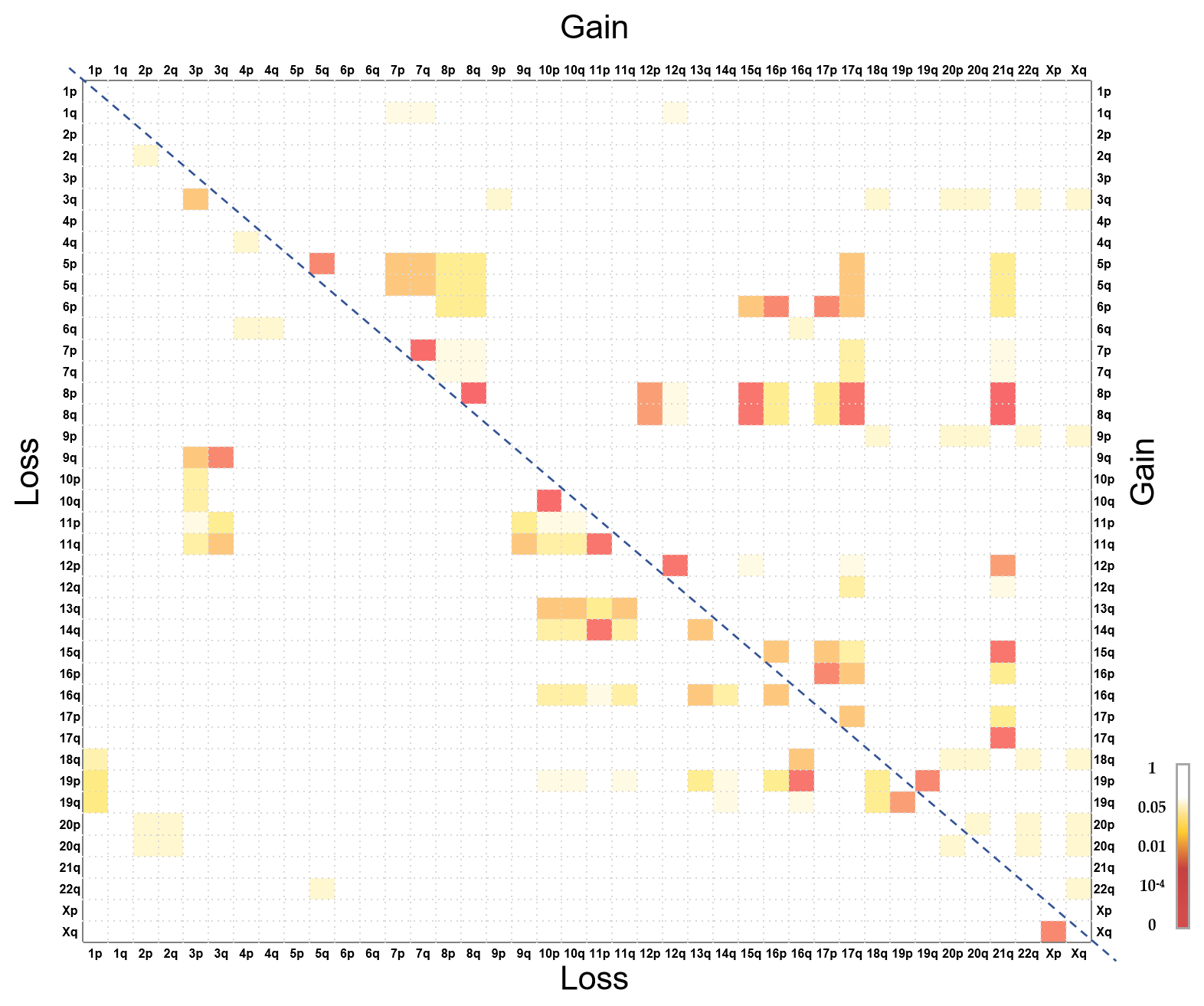

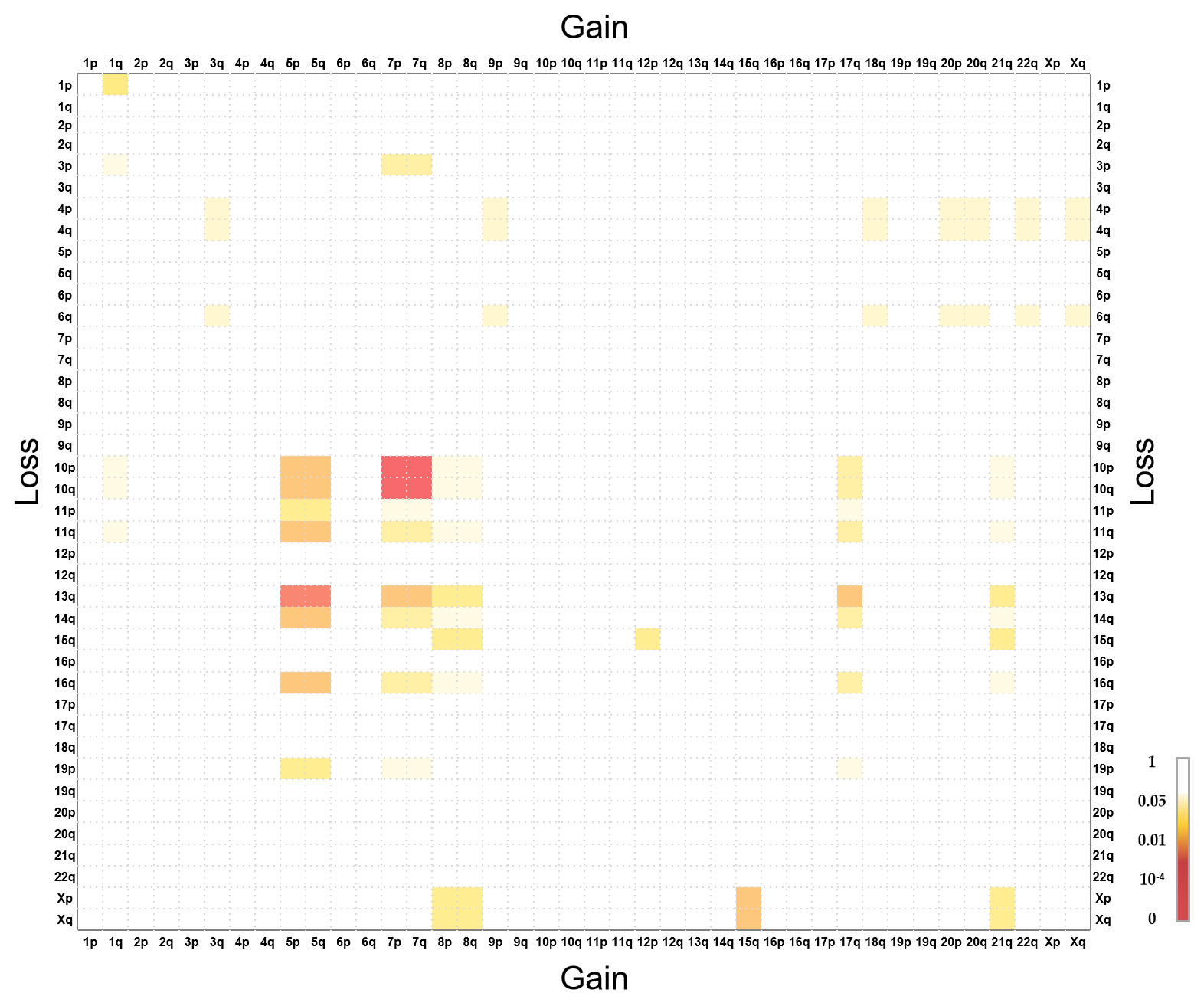


**Supplementary Figure S13.** Fisher's exact test was used to calculate the co - occurrence probability of loss - loss and gain - gain events on the same chromosomal arm in (a) neuroblastomas, (c) rhabdomyosarcomas and (e) non-rhabdomyosarcomas.The area below the dashed line on the left represents loss - loss, while the area above the dashed line on the right represents gain - gain. Fisher's exact test was used to calculate the co - occurrence probability of loss - gain events on the same chromosomal arm in (b) neuroblastomas, (d) rhabdomyosarcomas and (f) non-rhabdomyosarcomas. Areas filled with color represent p - values less than 0.05, and the darker the color, the smaller the p - value.

a b

9

7

3 4

Guidelines

(43.48%)

OncoKB

(47.82%)

NB

13

9

5 2

Guidelines

(48.28%)

OncoKB

(37.93%)

Non-RMS

**Supplementary Figure S14.** Clinically actionable variants in (a) neuroblastomas and (b) non-rhabdomyosarcomas. "Guidelines" indicates that the gene is included in the NCCN guidelines for the corresponding tumor type, while "OncoKB" indicates that the mutation site can be matched with targeted therapy information in the OncoKB database. The numbers in the figure represent the number of cases that match this area, with the proportion of cases detected in this category shown in parentheses.
